# Increase in the apparent intercalation ability of a platinum complex *via* multivalency by installation into the sidechain of a graft copolymer and observation of structural changes in the intercalated DNA†

**DOI:** 10.1039/c9ra03485d

**Published:** 2019-08-22

**Authors:** Shigehito Osawa, Riichi Takahashi, Remi Watanabe, Sayaka Kubo, Hidenori Otsuka

**Affiliations:** Department of Applied Chemistry, Faculty of Science Division I, Tokyo University of Science 1-3 Kagurazaka, Shinjuku-ku Tokyo 162-8601 Japan osawa-s@rs.tus.ac.jp h.otsuka@rs.kagu.tus.ac.jp; Graduate School of Science, Tokyo University of Science 1-3 Kagurazaka, Shinjuku-ku Tokyo 162-8601 Japan; Water Frontier Science and Technology Research Center, Research Institute for Science and Technology, Tokyo University of Science 1-3 Kagurazaka, Shinjuku-ku Tokyo 162-8601 Japan

## Abstract

Metal complexes with planar structures have been utilized as DNA intercalators that can be inserted into the base pairs of DNA strands, and have potential applications in DNA-targeting drug therapies. When designing the intercalator metal complexes, controlling their interactions with DNA is important, and has been performed by modifying the chemical structure of the metal ligand. Herein, we designed a graft copolymer segment having Pt complexes with bipyridine and poly(ethylene glycol) (p(PEGMA-*co*-BPyMA-Pt)) as another strategy to control the interaction with DNA *via* a multivalent effect. The p(PEGMA-*co*-BPyMA-Pt) increased not only the binding constant as one macromolecule but also the apparent binding constant per intercalator unit compared to the Pt complex with bipyridine (BPy-Pt). Moreover, p(PEGMA-*co*-BPyMA-Pt) induced a larger change in DNA structure using lower amounts of Pt than BPy-Pt. These observed properties of p(PEGMA-*co*-BPyMA-Pt) suggest that grafting intercalators on polymer segments is a promising approach for designing novel types of intercalators.

## Abstract 2

Pt complexes increase their apparent binding constant by grafting on sidechains of polymer segments *via* multivalent effect.

## Graphical abstract

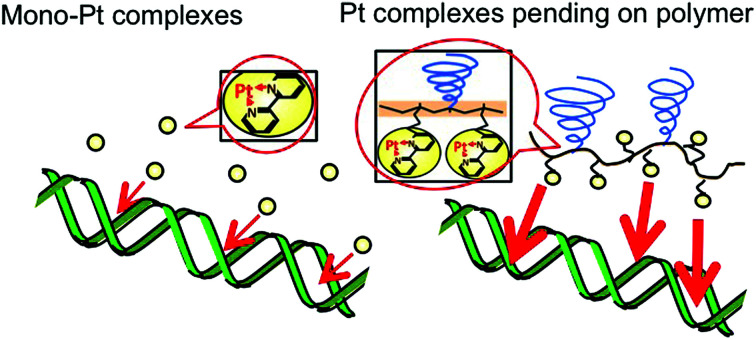

## Introduction

Metal complexes with planar structures have been widely known as DNA intercalators that can be inserted between a base pair of DNA.^1–4^ The intercalator metal complexes change the DNA structure and regulate gene expression.^5,6^ Owing to their ability to target DNA, these metal complexes have been incorporated into drugs, including anticancer drugs.^2,3,7^ With this form of drug, it is important to control the interaction between the drug and the DNA because this parameter is correlated to medicinal efficacy, suppression of side effects, and drug dosage. There are several strategies to increase binding affinity to DNA *via* enhancing electrostatic, hydrogen bonding, entropic, van der Waals, and hydrophobic interactions.^6–9^ Herein, we proposed the preparation of polymer grafted metal complexes as another approach to control the interaction without changing the structure of intercalating moieties, which can be a widely applicable strategy for various metal ligands. The polymer grafting intercalator is expected to show not only an increased binding constant as one macromolecule, but also an increased apparent binding constant per intercalator unit by a multivalent effect. The multivalent effect has been studied for designing drugs with high targeting properties,^10,11^ drug delivery systems^12,13^ and sensitive biosensors.^14^ In this study, we evaluated the increase in binding affinity per intercalator unit of the polymer for DNA binding and, furthermore, investigated the behaviour of structural changes in intercalated DNA.

**Scheme 1:**
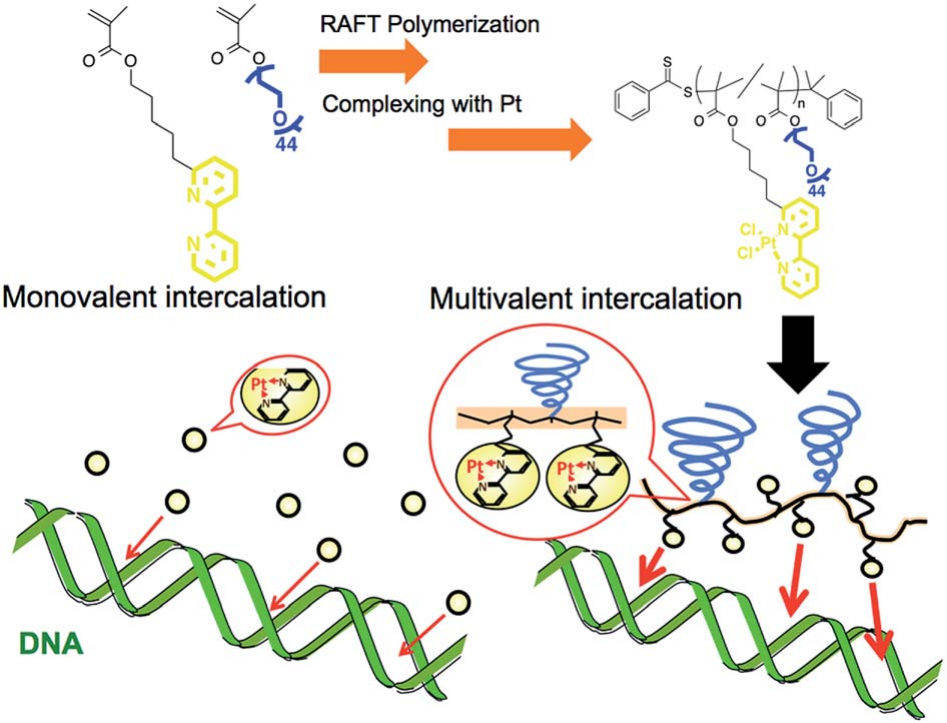
Schematic illustration of polymerized platinum complex and its multivalent effect.

Polymerized metal complexes prepared through the formation of coordinate bonds by mixing metal and ligand monomers have been reported in the literature.^15,16^ Compared to these types, it is more feasible to evaluate the required intercalator density in the polymer chain for the multivalency by polymer grafting the ligand units into a sidechain. This is because the number of intercalators per polymer chain can be controlled by adjusting the metal/polymer molar ratio without changing the main chain of the polymer segment. This investigation may be further feasible when the formation of metal complex is quantitative to the feed ratio of the metal/polymer, which requires the ligand forming enough stable coordinate bonds with metals. In this study, 2,2′-bipyridine (BPy) group was selected as the ligand grafting on the polymer chain because it can form a stable coordinate bond with Pt, and the resulting complex has a planar structure capable of intercalating with DNA strands.^17,18^ Considering that the alkyl chain in the *ortho*- or *para*-position on the pyridine ring may stabilize the coordinate bond by donating an electron to the nitrogen,^18^ here we designed 6-[5-pentan-1-methacrylate]-2,2′-bipyridine (BPyMA) as the BPy monomer. The alkyl chain of pentene in the monomer functions as the spacer between the main polymer chain and the BPy-Pt group, and existence of the spacer contributes to an increase in conformational number and accessibility of BPy-Pt units to DNA strands. In addition, polyethyleneglycol-methacrylate (PEGMA) was copolymerized to increase aqueous solubility of the polymer and to suppress intermolecular chelating of Pt, which may induce gelation of the components,^19^ by increasing steric repulsion among polymer chains (Scheme 1).

## Materials and methods

### Materials

The graft copolymer of BPyMA and PEGMA (*M*_n_ = 2089 Da) (p(PEGMA-*co*-BPyMA)) was prepared by reversible addition-fragmentation chain transfer (RAFT) polymerization (see ESI 2 and 3† for detailed preparation methods). The polymer grafting Pt complexes were prepared by mixing p(PEGMA-*co*-BPyMA) with DMSO-Pt in methanol (see ESI 3 and 4† for detailed preparation methods). Ethidium bromide (EtBr) solution (10 mg mL^−1^) (Wako Pure Chemical Co., Osaka, Japan), deoxyribonucleic acid sodium salt from calf thymus (CT-DNA) (Sigma-Aldrich Japan, Tokyo, Japan), plasmid DNA (pDNA) of pBR322 DNA (Nippon gene, Tokyo, Japan) and 6× loading dye (Funakoshi, Japan, Tokyo) were used as supplied by the manufacturer.

### Evaluation on binding constant to DNA

BPy-Pt and p(PEGMA-*co*-BPyMA-Pt) were dissolved in DMSO to adjust BPy-Pt concentration to 10 mM. In the case of p(PEGMA-*co*-BPyMA-Pt), 16.2 mg of p(PEGMA-*co*-BPyMA-Pt) was dissolved in 1 mL of DMSO considering p(PEGMA-*co*-BPyMA-Pt) has molar composition PEGMA/BPyMA-Pt of 14/27. Separately from these solutions, PBS solutions containing 4 μM of EtBr, 0.0117 mg mL^−1^ of CT-DNA was prepared. Then, DMSO and 10 mM sample solutions were added to 2463 μL of the PBS solutions as described in Table 1. Fluorescence intensities of the prepared mixtures at 580 nm, with excitation at 510 nm, were measured after 24 hours using Jasco FP-6500 (JASCO Co., Tokyo, Japan). Binding constants of BPy-Pt and p(PEGMA-*co*-ByMA-Pt) were obtained from the following equation:*C*_EtBr_*K*_EtBr_ = *C*_50%_*K*_50%_*C*_EtBr_: concentration of EtBr, *K*_EtBr_: binding constant of EtBr, concentration of ligand required for decreasing 50% fluorescence intensity derived from EtBr-DNA complex, *K*_b_: binding constant of intercalator.

**Table 1:** The solutions added to the PBS solutions containing EtBr and CT-DNA

Sample solution (μL)	DMSO (μL)	Final concentration of BPy-Pt (μM)
0	37.4	0
1	36.4	4
3	34.4	12
5	32.4	20
7	30.4	28
9	28.4	36
10	27.4	40
15	22.4	60
20	17.4	80

As for the binding test of the p(PEGMA-*co*-BPyMA-Pt/BPyMA) complexes with BPyMA-Pt/BPyMA ratios of 23/77, 50/50 and 67/33, DMSO solutions containing 10 mM of BPy-Pt units were first prepared and used in the same manner as the p(PEGMA-*co*-BPyMA-Pt).

BPy and p(PEGMA-*co*-BPyMA) were also dissolved in DMSO and the concentration of BPy units was adjusted to 10 mM. Binding ability of BPy and p(PEGMA-*co*-BPyMA) were also evaluated in the same manner as above (ESI 7†).

### Evaluation of the intercalated DNA with circular dichroism (CD) spectra measurement

BPy-Pt and p(PEGMA-*co*-BPyMA-Pt) were dissolved in DMSO to adjust the BPy-Pt concentration to 10 mM. These prepared solutions and DMSO were mixed as described in Table 2 and added to 9.85 mL of separately prepared 0.070 mg mL^−1^ CT-DNA solutions in PBS. After 24 hours incubation, CD spectra of the samples were obtained using CD JASCO J-725 (JASCO Co., Tokyo, Japan) with wavelength range from 235 to 350 nm, scanning speed of 200 nm min^−1^, and data processing three times every 1 nm interval.

**Table 2:** The solutions added to the CT-DNA PBS solutions

Sample solutions (μL)	DMSO (μL)	Conc. of BPy-Pt units (μM)	Molar ratio of BPy-Pt/base (Pt/P)
0	150	0	0
14.2	135.8	14.2	0.063
28.3	121.7	28.3	0.13
42.5	107.5	42.5	0.19
58.9	91.1	58.9	0.25

### Observation of plasmid DNA (pDNA) incubated with the intercalators by gel electrophoresis

BPy-Pt and p(PEGMA-*co*-BPyMA-Pt) were dissolved in DMSO to adjust the BPy-Pt concentration to 10 mM. Separately, PBS solution containing 0.3 μg μL^−1^ pDNA was prepared. To vary the mixing ratio of pDNA and intercalators, a series of BPy-Pt and p(PEGMA-*co*-BPyMA-Pt) sample solutions with varying BPy-Pt concentrations was prepared by diluting the above prepared 10 mM sample solutions with DMSO as shown in Table 3 and then, 9850 μL of deionized water was added. Twelve microliters of the sample solutions were mixed with 3 μL of pDNA solutions, which contain 2.77 nmol of base units, and incubated for 24 hours. Then, 3 μL of 6× loading dye were added to the sample solutions and 12 μL of the sample solutions were loaded onto 0.9% agarose gels. After gel electrophoresis at 50 V for 90 min, pDNA on the gel was visualized by soaking the gel in 0.5 mg mL^−1^ of EtBr solution and images were captured with GEL DOC EZ Imager (BIO-RAD Lab. Inc., PA).

**Table 3:** Dilution of sample solutions for adjusting final Pt/P^a^ values after adding to the pDNA solution

Intended Pt/P^a^ after mixing with pDNA	Sample solutions (μL)	DMSO (μL)
0.01	2.31	147.8
0.03	6.92	143.0
0.05	11.5	138.5
0.07	16.2	133.8
0.09	20.8	129.2
0.11	25.4	124.6
0.13	30.0	120.0
0.15	34.6	115.4
0.17	39.2	110.8
0.19	43.8	106.2
0.21	48.5	101.5
0.23	53.1	96.9

a Molar ratio of Pt and phosphates (P) on pDNA.

### Observation of pDNA incubated with the intercalators by atomic force microscopy (AFM)

The above prepared mixture of BPy-Pt or p(PEGMA-*co*-BPyMA-Pt) and pDNA at BPy-Pt/phosphates molar ratio (Pt/P) = 0, 0.07, 0.17 and 0.23 were diluted with deionized water 10 times. The samples were fixed on mica substrates pre-treated with MgCl_2_. AFM imaging was performed with the MFP-3D-BIO-J (Oxford Instrument, UK, Abingdon) in AC mode, with cantilever of 300 kHz resonance frequency and 42 N m^−1^ spring constant (OLYMPUS, Japan, Tokyo).

## Results and discussion

The BPyMA monomer was prepared by 7-step chemical reactions as described in ESI (see ESI 2†) and the products in each step were confirmed by ^1^H-NMR. The obtained BPyMA was co-polymerized with PEGMA *via* RAFT polymerization using cumyl dithiobenzoate (CDB) as a chain transfer agent and 2,2′-azobis(2-methylpropionitrile) (AIBN) as a radical initiator (ESI 3†).^20^ Composition of the PEGMA/BPyMA ratio in the obtained polymer was calculated to be 14/27 by monomer conversion study with ^1^H-NMR, which is close in value to the monomer composition in the initial polymerization mixture. The gel permeation chromatography (GPC) measurement of the obtained polymer showed a unimodal and symmetric peak with *M*_w_/*M*_n_ = 1.29 in the PEG standard (Fig. S5b†), suggesting RAFT polymerization was performed in well-controlled manner. Under the assumption that the RAFT polymerization successfully proceeded, molecular weight was calculated to be 38 900 Da from the 46% monomers conversion determined by ^1^H-NMR (Fig. S3†). The comparable apparent molecular weight value, 33 520 Da, was also obtained by static light-scattering measurements (Fig. S6†), strongly supporting successful preparation of p(PEGMA-*co*-BPyMA) *via* RAFT polymerization.

The Pt complex of the obtained p(PEGMA-*co*-BPyMA) was prepared in a previously reported manner (ESI 4-1†)^17,21^. Formation of the Pt complexes was evaluated by observing the ^1^H-NMR peak shift of proton signals on the 6 and 6′ positions of the bipyridyl group from 8.7 ppm to 9.9 ppm (Fig. S10†). Changing feeds of Pt to p(PEGMA-*co*-BPyMA) showed the different peak intensity ratios of 23/77, 50/50, 67/33, and 100/0 at 8.7 ppm and 9.9 ppm, all consistent with the mixing molar ratio of Pt and BPyMA units (Table S7†). These results are indicative of successful preparation of the p(PEGMA-*co*-BPyMA-Pt/BPyMA) series, and chelation of one Pt with one BPy unit without intra- or inter-polymer chelating.

**Fig. 1:**
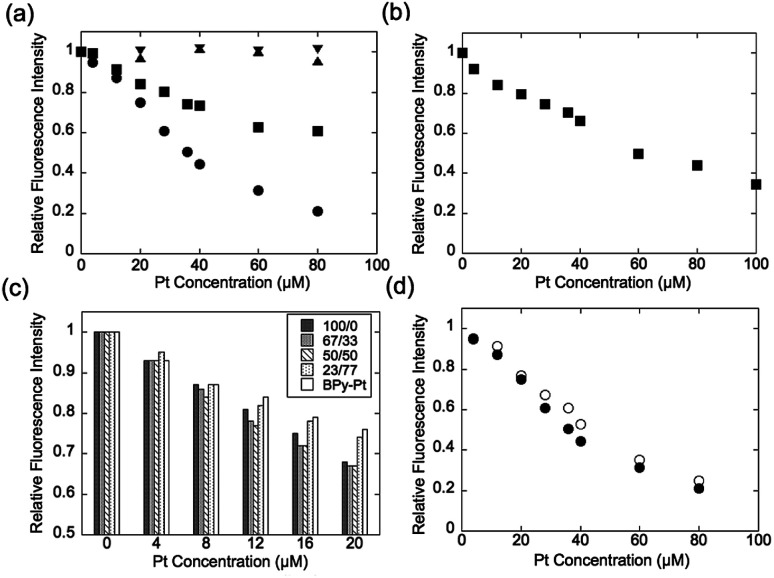
EtBr exclusion assays. Tracking fluorescence intensity change of (a) 4 μM EtBr solution containing 0.0117 mg mL^−1^ of CT-DNA with escalating addition of BPy (▲), p(PEGMA-*co*-BPyMA) (▼), BPy-Pt (■), and p(PEGMA-*co*-BPyMA-Pt) (●), (b) 2 μM EtBr solution containing 0.00585 mg mL^−1^ of CT-DNA with escalating addition amount of BPy-Pt, (c) 4 μM EtBr solution containing 0.0117 mg mL^−1^ of CT-DNA with escalating addition of Pt complex with series of p(PEGMA-*co*-ByMA-Pt/ByMA) at BPyMA-Pt/BPyMA ratio of 23/77, 50/50, 67/33 and 100/0, and (d) 4 μM EtBr solution containing 0.0117 mg mL^−1^ of CT-DNA with escalating addition of p(PEGMA-*co*-BPyMA-Pt/BPyMA) at BPyMA-Pt/BPyMA ratio of 50/50 (○) and 100/0 (●).

Expression of the multivalent effect was evaluated from comparison between apparent binding constant of p(PEGMA-*co*-BPyMA) (BPyMA-Pt/BPyMA = 100/0) per one BPy-Pt unit to DNA and that of BPy-Pt monomer. The binding constants to DNA were investigated by an ethidium bromide (EtBr) exclusion assay, which tested the EtBr binding capacity to DNA against escalating concentration of BPy-Pt. EtBr shows a strong fluorescence at 580 nm when it intercalates with DNA. Thus, the binding constant of BPy-Pt to DNA can be evaluated by tracking fluorescence intensity at 580 nm against the escalated concentration of BPy-Pt components. p(PEGMA-*co*-BPyMA-Pt) more largely decreased the fluorescence intensity than BPy-Pt (Fig. 1a), although p(PEGMA-*co*-BPyMA) without the Pt complex did not show any significant decrease in fluorescence intensity, suggesting intercalating ability per BPy-Pt was enhanced by constructing polymers of p(PEGMA-*co*-BPyMA-Pt). The binding constant (*K*_b_) of p(PEGMA-*co*-BPyMA-Pt) was calculated by the following equations as previously reported;^22^*K*_EtBr_*C*_EtBr_ = *K*_b_*C*_50%_, where *K*_EtBr_ and *C*_EtBr_ describe the binding constant of EtBr to DNA and EtBr concentration, respectively, and *C*_50%_ is the concentration of BPy-Pt required for 50% decrease in EtBr-DNA fluorescence intensity. *K*_EtBr_ was calculated to be 6.26 × 10^5^ M^−1^ by making Schatchard's plots (see ESI 5†). *C*_EtBr_ of this solution was set to 4 μM, which saturated 0.0117 mg mL^−1^ of DNA solution (See ESI 6†). *C*_50%_ of p(PEGMA-*co*-BPyMA-Pt) was predicted to be 36 μM from the plots of fluorescence intensities of DNA-EtBr against the BPy-Pt (Fig. 1a). From these values and the above equation, *K*_b_ of p(PEGMA-*co*-BPyMA-Pt) was calculated to be 6.95 × 10^4^ M^−1^. In contrast, *K*_b_ of BPy-Pt to DNA cannot be obtained in the same experimental condition as p(PEGMA-*co*-BPyMA-Pt); the aqueous solubility of BPy-Pt is low and concentration of BPy-Pt cannot be increased to reduce 50% fluorescence of the 4 μM EtBr and 0.0117 mg mL^−1^ DNA mixture. Thus, *K*_b_ of BPy-Pt was evaluated using a solution containing 2 μM of EtBr (*C*_EtBr_) and 0.00585 mg mL^−1^ of DNA, which would require half the concentration of BPy-Pt as the above condition for reducing 50% of the fluorescence. *C*_50%_ was predicted to be 60.0 μM (Fig. 1b) and the *K*_b_ of BPy-Pt monomer was calculated to be 2.09 × 10^4^ M^−1^. This indicates the apparent binding constant of p(PEGMA-*co*-BPyMA-Pt) per one BPy-Pt unit was 3.3 times higher than that of the BPy-Pt monomer, suggesting that polymerization of BPy-Pt units induced a multivalent effect on the DNA intercalation. It is worth noting the binding constant of p(PEGMA-*co*-BPyMA-Pt) polymer chain as a macromolecule was calculated to be 1.87 × 10^6^ M^−1^, almost 100 times higher than that of BPy-Pt, under the assumption that p(PEGMA-*co*-BPyMA-Pt) has 27 BPyMA-Pt monomer units as shown in the ^1^H-NMR measurement (Fig. S3†). This also suggests that polymerizing intercalators have great potential to increase the binding constant to the DNA.

To further investigate the increased binding ability of the graft polymer complex, EtBr exclusion abilities of p(PEGMA-*co*-BPyMA-Pt/BPyMA) with differing Pt complex numbers were evaluated (Fig. 1c). p(PEGMA-*co*-BPyMA-Pt/BPyMA) with a complex ratio of 23/77 showed a similar trend as the BPy-Pt monomer, suggesting the 23/77 complex ratio may not increase the binding constant per BPy-Pt unit. In contrast, p(PEGMA-*co*-BPyMA-Pt/BPyMA) with a BPyMA-Pt/BPyMA ratio above 50/50 showed a reduction in the intensity of the fluorescence compared with the BPy-Pt monomer. This result indicated increasing the apparent binding ability per BPy-Pt unit required introduction of BPy-Pt units into more than one third of sidechains on p(PEGMA-*co*-BPyMA); approximately, p(PEGMA-*co*-BPyMA) has PEGMA components at one-third of the sidechains and BPyMA components at two-thirds of the sidechains. Thus, p(PEGMA-*co*-BPyMA-Pt/BPyMA) with BPyMA-Pt/BPyMA ratio of 50/50 have the PEGMA/BPyMA/BPyMA-Pt molar ratio of 1/1/1. EtBr exclusion capacities were comparable among p(PEGMA-*co*-BPyMA-Pt/BPyMA) complexes with BPyMA-Pt/BPyMA ratios of 50/50, 67/33, 100/0 (Fig. 1c). Indeed, 50/50 showed the binding constant per BPy-Pt unit of 6.30 × 10^4^ M^−1^, comparable to the binding constant of the 100/0 ratio (Fig. 1d). Under the assumption that p(PEGMA-*co*-BPyMA-Pt/BPyMA) has random architecture, the fraction of neighbouring BPyMA-Pt units in polymer sidechain increases when the BPyMA-Pt/BPyMA ratio escalates above 50/50. These data suggest that neighbouring BPyMA-Pt units may not contribute to enhance the binding ability per BPy-Pt of this polymer. Note that distance between ligands and special arrangements of them have a large influence on multivalent binding to the receptor.^11^ Thus, there are two speculations for explaining this behaviour: one is that the distance between BPy-Pt moieties on the sidechains of neighbouring monomer units does not match the distance between binding sites on DNA strands, and the other is that BPy-Pt on the neighbouring sidechains are not positioned to allow for binding to DNA strands due to steric hindrance between the sidechains. The observed increase in binding constant can likely be controlled by changing alkyl chain length between BPy group and main-chain, or polymerization group of monomer structure to change polymer tacticity and conformations, similar to that of the ligand–receptor multivalent studies.^11–14,17,18^ This will be the strategy for controlling binding ability of intercalator grafts on the polymer chain in future studies.

**Fig. 2:**
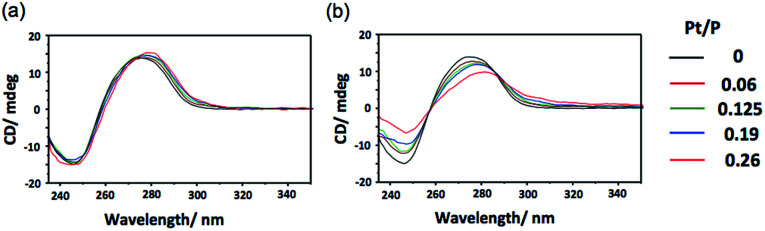
CD spectra of CT-DNA incubated with (a) BPy-Pt and (b) p(PEGMA-*co*-BPyMA-Pt) at various Pt/P ratios.

In addition to binding ability, the ability to induce structural change in DNA is also an important property of an intercalating drug.^5^ To investigate the polymerization effect on the DNA structural change, circular dichroism (CD) spectral measurements were performed after binding p(PEGMA-*co*-BPyMA-Pt) or BPy-Pt monomers to DNA at various mixing ratios of Pt/P, where Pt is the feeding molarity of platinum and P is that of phosphate in DNA. Note that BPy and p(PEGMA-*co*-BPyMA), before forming metal complexes with Pt, do not show specific spectra and do not change the spectra of DNA in CD measurements (Fig. S16a and b†). The BPy-Pt monomer showed a slight shift of the peak top (Fig. 2a), indicating BPy-Pt rewinds the DNA strand.^23^ In contrast, p(PEGMA-*co*-BPyMA-Pt) showed not only peak top shift but also a significant decrease in the negative band at 247 nm and positive band at 280 nm (Fig. 2b). Polymerization of BPy-Pt may enhance the ability the change the double-helix structure of intercalated DNA.

**Fig. 3:**
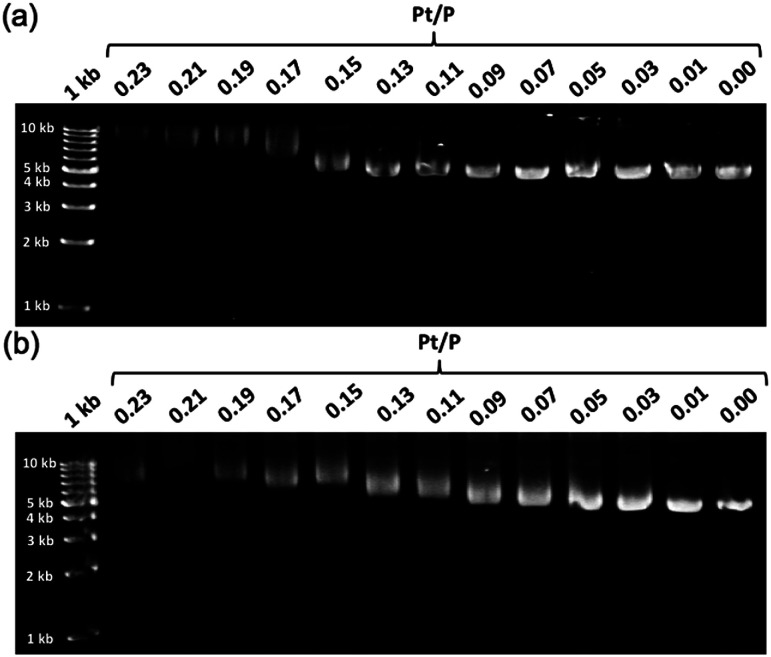
Gel images of pDNA incubated with escalating concentration of (a) BPy-Pt and (b) p(PEGMA-co-BPyMA-Pt).

**Fig. 4:**
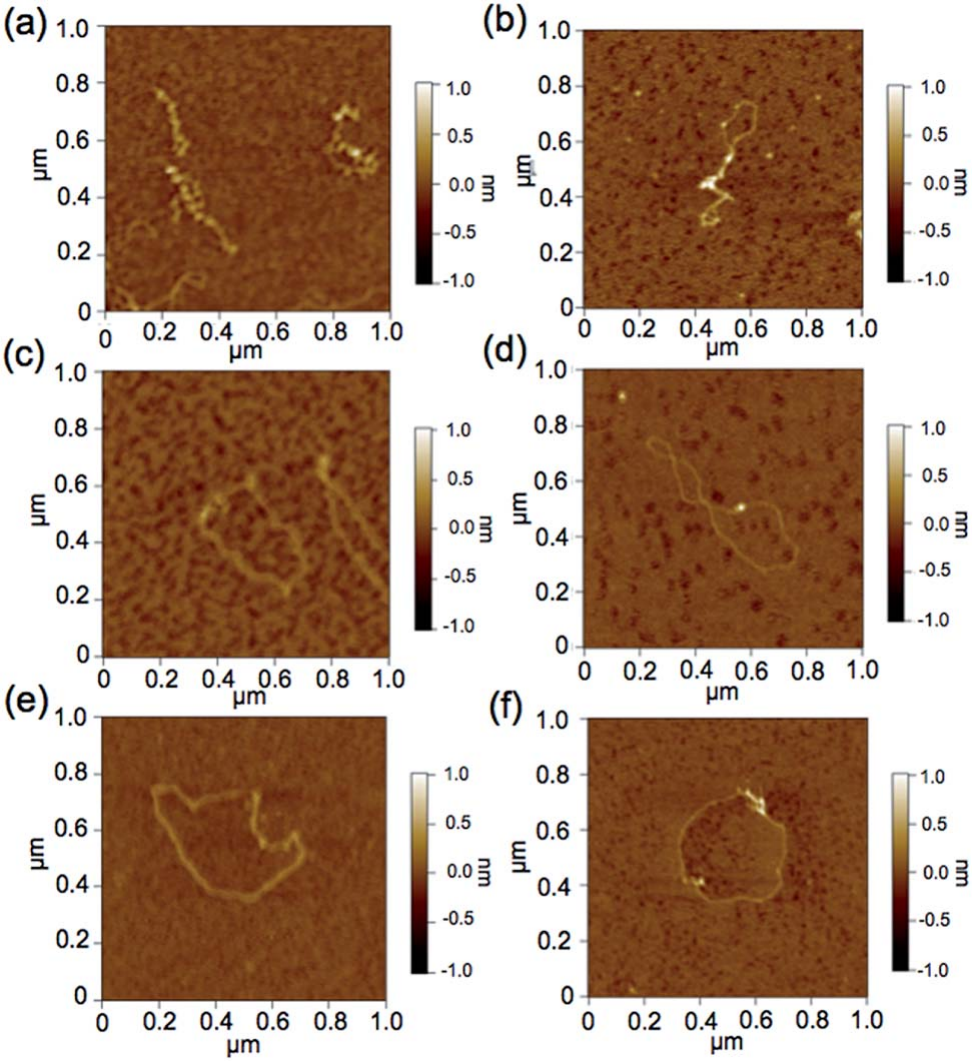
Representative micrographs of pDNA incubated with BPy-Pt at feeding ratio of Pt/P = (a) 0.07, (c) 0.17 and (e) 0.23, and incubated with p(PEGMA-*co*-BPyMA-Pt) at feeding ratio of Pt/P = (b) 0.07, (d) 0.17 and (f) 0.23.

To further demonstrate the influence of the graft copolymer on the morphology of intercalated DNA, plasmid DNA (pDNA), which has a supercoil structure, was incubated with various concentrations of BPy-Pt monomers and p(PEGMA-*co*-BPyMA-Pt), then, DNA band shift was observed by gel electrophoresis (Fig. 3a and b). DNA samples with the complete band shift (Pt/P = 0.23) showed open circular DNA as major structure (Fig. 4e, f, S17d and g†), suggesting that the observed band shift in gel electrophoresis may reflect pDNA structural change from the supercoil to the open circular form. p(PEGMA-*co*-BPyMA-Pt) started to shift the DNA bands at Pt/P = 0.07, a significantly lower concentration compared to the BPy-Pt monomer. Interestingly, the band shift pattern is quite different between p(PEGMA-*co*-BPyMA-Pt) and BPy-Pt. In the case of p(PEGMA-*co*-BPyMA-Pt), DNA bands appeared to gradually shift to the open circular form as p(PEGMA-*co*-BPyMA-Pt) increased (Fig. 3b). In contrast, BPy-Pt shifted the DNA bands more sharply at Pt/P ratio above 0.13 (Fig. 3a). p(PEGMA-*co*-BPyMA-Pt), which can bind to DNA strands in a multivalent arrangement, may partially induce the DNA rewinding and change the supercoil structure because intercalating sites on DNA strands are locally concentrated. On the other hand, BPy-Pt monomers intercalated with the DNA strand in a random manner, and the pDNA supercoil structure was sustained at Pt/P ratios below 0.13. This is likely because the BPy-Pt intercalating sites on the DNA strands were not close enough to induce morphological changes in the supercoil. It is speculated that a rapid change from the supercoil to the open circular form is caused by increasing the Pt/P ratio above 0.15 because the distance between the intercalating sites become close enough. AFM showed consistent images of these gel electrophoresis results (Fig. 4a–d, S17 b, c, e and f†). These observed differences between p(PEGMA-*co*-BPyMA-Pt) and BPy-Pt monomers suggest that the construction of the graft copolymer can affect the three-dimensional structural changes in intercalated DNA.

## Conclusions

In conclusion, installing BPy-Pt units into the sidechain of the graft copolymer increased the binding constant to DNA due to the multivalent effect, and induced larger structural changes to the double-helix structure of the intercalated DNA compared to the original BPy-Pt. Moreover, polymerizing BPy-Pt influenced the structural changes in supercoiled pDNA induced by binding, suggesting potential for controlling the three-dimensional structure of DNA. The observed physicochemical properties in this study are important for designing intercalating molecules, including DNA-targeting drugs.

## Conflicts of interest

There are no conflicts to declare.

## Supplementary Material

RA-009-C9RA03485D-s001
