# Supplementary material for: Increase in the apparent intercalation ability of a platinum complex via multivalency by installation into the sidechain of a graft copolymer and observation of structural changes in the intercalated DNA
Source: RSC Adv. 2019 Aug 22;9(45):26429–34. doi: 10.1039/c9ra03485d (PMC9070401; doi:10.1039/c9ra03485d)
Supplement: RA-009-C9RA03485D-s001 [file RA-009-C9RA03485D-s001.pdf]

Electronic Supplementary Material (ESI) for RSC Advances.
This journal is © The Royal Society of Chemistry 2019

**SI.1 Materials**

2,6-Dibromopyridine (Tokyo Kasei Kogyo Co. Ltd., Tokyo, Japan), 2-Pyridylzinc bromide (Sigma-Aldrich Japan, Tokyo, Japan), Tetrakis(triphenylphosphine) palladium(0) ($Pd(PPh_3)_4$) (Tokyo Kasei Kogyo Co. Ltd., Tokyo, Japan), Copper(I) Iodide (Kanto Chemical Co., Inc., Tokyo, Japan), N,N'-Dimethyl cyclohexane 1,2-diamine (Sigma-Aldrich Japan, Tokyo, Japan), Sodium lodide (Wako Pure Chemical Co., Osaka, Japan), 4-Pentyn-1-ol (Wako Pure Chemical Co., Osaka, Japan), 3,4-Dihydro-2H-pyran (Wako Pure Chemical Co., Osaka, Japan), p-Toluenesulfonic acid monohydrate ($TsOH-H_2O$) (Wako Pure Chemical Co., Osaka, Japan), Bis(triphenylphosphine)palladium(II) Dichloride ($Pd(PPh_3)_2Cl_2$) (Tokyo Kasei Kogyo Co. Ltd., Tokyo, Japan), Diisopropylamine (Wako Pure Chemical Co., Osaka, Japan), Palladium-Activated Carbon (Pd 10%) (Wako Pure Chemical Co., Osaka, Japan), Methacrylic anhydride 94% (Wako Pure Chemical Co., Osaka, Japan), Triethylamine (Wako Pure Chemical Co., Osaka, Japan), Bromobenzene (Wako Pure Chemical Co., Osaka, Japan), Magnesium (Wako Pure Chemical Co., Osaka, Japan), Carbon disulfide (Wako Pure Chemical Co., Osaka, Japan), α-Methylstyrene (Sigma-Aldrich Japan, Tokyo, Japan), 2,2'-Azobisisobutyronitrile (AIBN) (Wako Pure Chemical Co., Osaka, Japan), Poly(ethylene glycol)monomethylethermethacrylate (MeO-PEG-MA) (50wt% in Water), Potassiumtetrachloroplatinate (II) (Sigma-Aldrich Japan, Tokyo, Japan), 2'2-Bipyridine (Wako Pure Chemical Co., Osaka, Japan), Tetrahydrofuran (THF) (Wako Pure Chemical Co., Osaka, Japan), Sodium hydrogen carbonate ($NaHCO_3$) (Wako Pure Chemical Co., Osaka, Japan), Sodium chloride (NaCl) (Wako Pure Chemical Co., Osaka, Japan), Chloroform ($CHCl_3$) (Sigma-Aldrich Japan, Tokyo, Japan), 1,4-Dioxane (Wako Pure Chemical Co., Osaka, Japan), Ammonia solution ($NH_3$aq) (Wako Pure Chemical Co., Osaka, Japan), Dichloromethane dehydrate (Wako Pure Chemical Co., Osaka, Japan), Hexane (Sigma-Aldrich Japan, Tokyo, Japan), Ethyl acetate (EtOAc) (Sigma-Aldrich Japan, Tokyo, Japan), Acetone (Sigma-Aldrich Japan, Tokyo, Japan), Hydrochloric acid (1N) (Wako Pure Chemical Co., Osaka, Japan), Carbon tetrachloride (Wako Pure Chemical Co., Osaka, Japan), Diethylether ($Et_2O$) (Sigma-Aldrich Japan, Tokyo, Japan), Chloroform ($CHCl_3$) (Sigma-Aldrich Japan, Tokyo, Japan), N,N'-dimethylformamide (DMF) (Wako Pure Chemical Co., Osaka, Japan), 2-Propanol (Wako Pure Chemical Co., Osaka, Japan), Benzene (Sigma-Aldrich Japan, Tokyo, Japan), Dimethyl Sulfoxide (DMSO) (Wako Pure Chemical Co., Osaka,

Japan), Ethanol (EtOH) (Wako Pure Chemical Co., Osaka, Japan), Potassium Chloride (Wako Pure Chemical Co., Osaka, Japan), Potassium Dihydrogen phosphate (Wako Pure Chemical Co., Osaka, Japan), Disodium hydrogenphosphate dodecahydrate (Sigma-Aldrich Japan, Tokyo, Japan), Sodium sulfate (Wako Pure Chemical Co., Osaka, Japan), Magnesium sulfate ($MgSO_4$) (Wako Pure Chemical Co., Osaka, Japan), Ethylenediamine-N,N,N',N'-tetraacetic acid disodium salt dehydrate (EDTA・2Na) (Wako Pure Chemical Co., Osaka, Japan), Silica gel 60 (MERCK Co., Tokyo, Japan), Sodium hydrogen carbonate (Wako Pure Chemical Co., Osaka, Japan), and Chloroform d-1 (MERCK Co., Tokyo, Japan), Ethidium Bromide (EtBr) solution (10 mg/mL) (Wako Pure Chemical Co., Osaka, Japan), and Deoxyribonucleic acid sodium salt from calf thymus (CT-DNA) (Sigma-Aldrich Japan, Tokyo, Japan).

## SI. 2 Synthesis of 6-(pentan-1-methacrylate)-2,2'-bipyridine (BPyMA) (9)

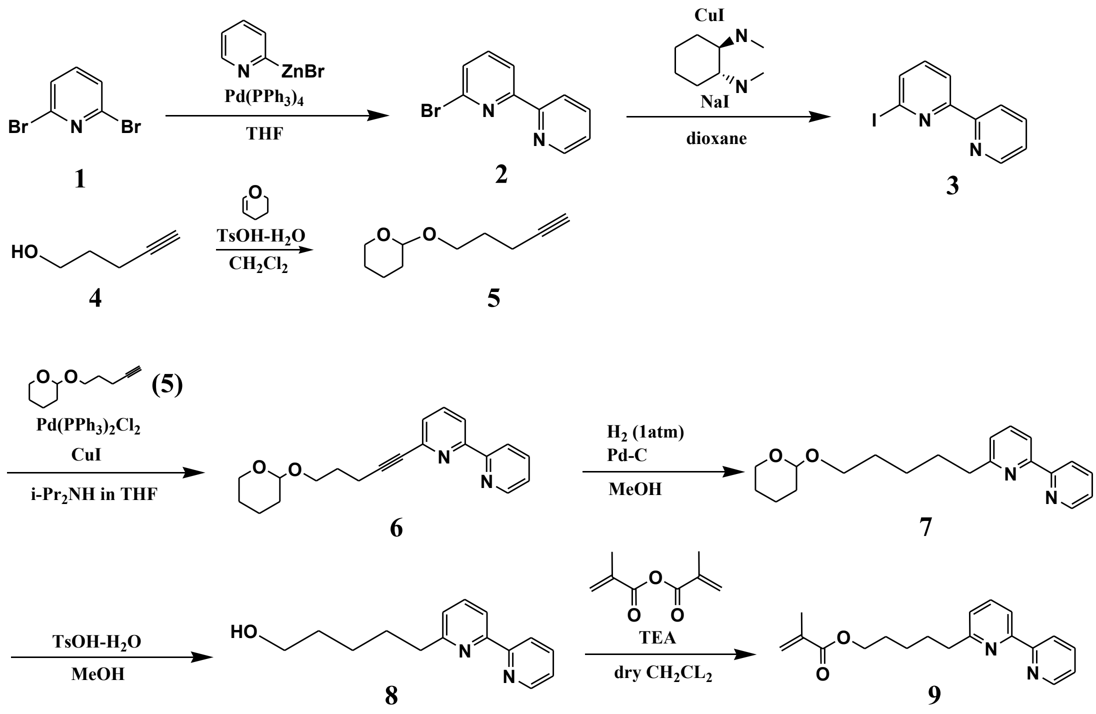


Scheme 1. Synthesis of (6-(pentan-1-methacrylate)-2,2'-bipyridine (BPyMA)

## SI. 2-1 Synthesis of 6-Bromo-2, 2'-bipyridine (2)

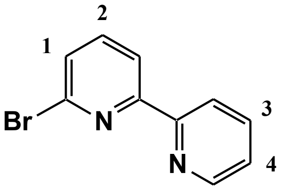


**2**

2,6-Dibromopyridine **(1)** (5.9 g, 25 mmol) and $Pd(PPh_3)_4$ (578 mg, 0.5 mmol) were dissolved into 50 mL of the THF solution with 0.5 M 2-pyridylzinc bromide, followed by stirring at r.t. overnight. This process was carried out under Ar atmosphere. Then, 300 mL of deionized water was added to the reaction mixture, and excess amount of EDTA・2Na and $Na_2CO_3$ were added to completely dissolve the precipitate. The product was extracted by ethyl acetate from the reactant. The product solution was dehydrated by magnesium sulfate and concentrated by evaporation. The obtained sample solution was further purified by silica column using ethyl acetate/hexane = 1/10 as a mobile phase, followed by condensation and drying in vacuum to obtain the product (yields: 2.93 g, 49%). The product is characterized by $^1$H-NMR (JEOLAL-500, 500 MHz,

$CDCl_3$) δ: 8.67(1 H, dq, J = 0.9, 4.9, **5**), 8.42-8.37(2 H, m), 7.82(1 H, td, J = 1.8, 7.8, **2**), 7.67(1 H, t, J = 7.8, **3**), 7.49(1 H, dd, J = 0.8, 7.8, **1**), 7.34-7.32 (1 H, m, **4**)

**SI. 2-2 Synthesis of 6-Iodo-2, 2'-bipyridine (3)**

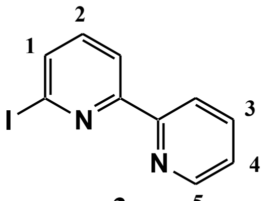


**3**

The obtained 6-Bromo-2, 2'-bipyridine **(2)** (2.93 g, 12.5 mmol), copper iodide (118.8 mg, 0.62 mmol), sodium iodide (3.75 g, 28.3 mmol), dimethyl-cyclohexane-1.2-diamine (213 mg, 1.5 mmol) were dissolved in 40 mL of 1,4-Dioxane under the Ar atmosphere, followed by reflux at 110 ºC for 24 hours. Then, 80 mL of 28% ammonia aqueous solution and 300 mL of water were added into the reaction solution, followed by extraction of the product by $CHCl_3$. The obtained product solution was dehydrated by magnesium sulfate and then the product was obtained by condensation and drying in vacuum. (yields: 3.53 g, 96%). The obtained product was characterized by $^1$H-NMR(JEOLAL-500, 500 MHz, $CDCl_3$) δ: 8.66(1 H, dq, J = 0.9, 4.6, **5**), 8.40-8.38(2 H, m), 7.81(1 H, td, J = 1.6, 7.6, **2**), 7.72(1 H, dd, J = 0.9, 7.6, **1**), 7.44(1 H, t, J = 7.8, **3**), 7.33-7.31(1 H, m, **4**)

**SI. 2-3 Synthesis of 2-Pent-4-ynloxy-tetrahydro-pyran (5)**

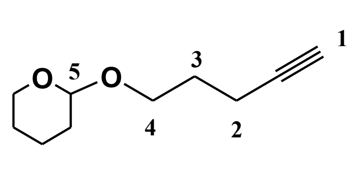


**5**

4-Pentyn-1-ol (2 g, 23 mmol), TsOH-$H_2O$ (44 mg, 0.23 mmol) were dissolved in 50 mL of dichloromethane on the ice and then, 3,4-dihydro-2H-pyran (2150 mg, 25.5 mmol) was slowly dropped into the above solution, followed by stirring on ice for an hour and at r.t. for another hour. The reaction solution was mixed with saturated

$NaHCO_3$ solution and the dichloromethane fraction was collected to extract the product. After dehydration by magnesium sulfate and condensation of the collected solution, the product solution was further purified by silica column using ethyl acetate/hexane = 1:20 as mobile phase. The product was obtained by condensation and drying in vacuum (yields: 2.80 g, 72%). The obtained product was characterized by $^1$H-NMR(JEOLAL-500, 500 MHz, $CDCl_3$) δ: 4.60(1 H, t, J = 4.1 Hz, **5**), 3.89-3.81(2 H, m, **4**), 3.53-3.46(2 H, m, **2**), 2.32-2.31(2 H, m, **3**), 1.95(1 H, t, J = 2.6 Hz, **1**), 1.85-1.78(3 H, m), 1.74-1.68(1 H, m), 1.62-1.50(4 H, m)

**SI. 2-4 Synthesis of 6-[5-(Tetrahydro-pyran-2-yloxy)-pent-1-ynyl]-2,2'-bipyridine (6)**

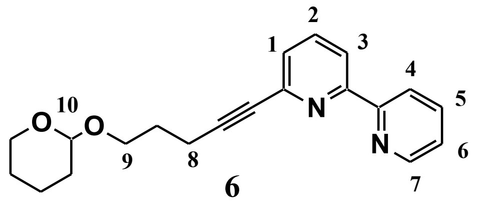


**6**

6-Iodo-2, 2'-bipyridine **(3)** (3.53 g, 12.5 mmol), 2-pent-4-ynloxy-tetrahydro-pyran **(5)** (2.1 g, 12.5 mmol), $Pd(PPh_3)_2Cl_2$ (578 mg, 0.5 mmol), copper iodide (190 mg, 1.0 mmol), diisopropylamine (4.97 g, 50 mmol) were dissolved in 100 mL of THF under Ar atmosphere and stirred overnight at r.t. Then, reactant was filtered to exclude precipitate and the supernatant was collected. After condensation in vacuum, the solution was added into the chloroform, mixed with brine to exclude the unreacted reagent and salt, and then, chloroform fraction was collected. After dehydration by magnesium sulfate and condensation of the collected solution, the product solution was further purified by silica column using ethyl acetate/hexane = 1:10 as mobile phase (yields: 2.15 g, 53%). $^1$H-NMR(500 MHz, $CDCl_3$) δ: 8.66(1 H, dq, J = 0.9, 4.9, **7**), 8.45(1 H,d, J = 7.9, **3**), 8.32(1 H, dd, J = 0.9, 7.9, **4**), 7.80(1 H, td, J = 1.8, 7.8, **2**), 7.75(1 H, t, J = 7.8, **5**), 7.40(1 H, dd, J = 0.9, 7.9, **1**), 7.31-30(1 H, m, 6), 4.64(1 H, t, J = 3.7, **10**), 3.92-3.89(2 H, m, 9), 3.59-3.51(2 H, m), 2.62(2 H, td, J = 1.6, 7.2, **8**), 1.99-1.94(2 H, m), 1.88-1.54(6 H, m)

## SI. 2-5 Synthesis of 6-[5-(Tetrahydro-pyran-2-yloxy)-pentyl]-2,2'-bipyridine (7)

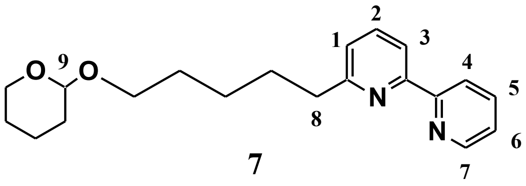

**7**

6-[5-(Tetrahydro-pyran-2-yloxy)-pent-1-ynyl]-2,2'-bipyridine **(6)** (2.15 g, 6.67 mmol) was dissolved in 70 mL of methanol and then, 400 mg of palladium-activated carbon (Pd 10%) was added. The reduction of alkyne was carried out under $H_2$ atmosphere by stirring at r.t. for 4 hours. The reaction solution was filtered to exclude palladium-activated carbon, followed by condensation and drying to obtain the product. (2.03 g, 93%). The obtained product was characterized by $^1$H-NMR (JEOLAL-500, 500 MHz, $CDCl_3$) δ: 8.67(1 H, dq, J = 0.8, 4.8, **7**), 8.44(1 H,d, J = 7.9, **3**), 8.18(1 H, dd, J = 0.8, 7.9, **4**), 7.82-7.79(1 H,,m, **2**), 7.71(1 H, t, J = 7.9, **5**), 7.30-7.27(1 H, m, **6**), 7.15(1 H, d, J = 7.9, **1**), 4.57(1 H, t, J = 3.7, **9**), 3.88-3.83(1 H, m), 3.76-3.75(1 H, m), 3.50-3.46(1 H, m), 3.42-3.40(1 H, m), 2.88(2 H, t, J = 7.8, **8**), 1.88-1.65(6H, m), 1.56-1.49(6 H, m)

## SI. 2-6 Synthesis of 6-[5-Pentan-1-ol]-2,2'-bipyridine (8)

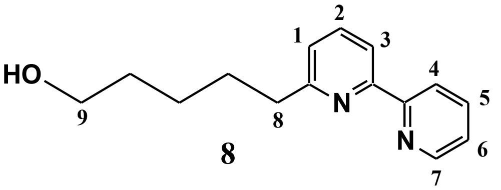

**8**

6-[5-(Tetrahydro-pyran-2-yloxy)-pentyl]-2,2'-bipyridine **(7)** (2.03 g, 6.22 mmol) and TsOH-$H_2O$ (1.77 g, 9.33 mmol) were dissolved in 70 mL of methanol and stirred at r.t. for 6 hours. The reactant was neutralized by adding 1 N NaOH solution and condensed by evaporation. Chloroform was added to the condensed product solution and then, unreacted reagent and salt were extracted by saturated $NaHCO_3$ solution and brine. The collected chloroform fraction was dehydrated by magnesium sulfate, condensed and dried in vacuum to recover the product (yields: 1.57 g). The obtained product was characterized by $^1$H-NMR(JEOLAL-500, 500 MHz, $CDCl_3$) δ: 8.67(1 H, dq, J = 0.9, 4.9, **7**), 8.43(1 H,d, J = 7.9, **3**), 8.18(1 H, dd, J = 0.9, 7.9, **4**), 7.82-7.81(1 H, m, **2**), 7.72(1 H, t, J = 7.6, **5**), 7.30-7.29(1 H, m, **6**), 7.16(1 H, d, J = 7.6, **1**), 3.67(2 H, q, J = 6.1, **9**),

2.83(2 H, t, J = 7.6, **8**), 1.88-1.85(2 H, m), 1.69-1.63(2 H, m), 1.50-1.47(2 H, m)

### SI. 2-7 Synthesis of 6-[5-Pentan-1-methacylate]-2,2'-bipyridine (BPyMA) (9)

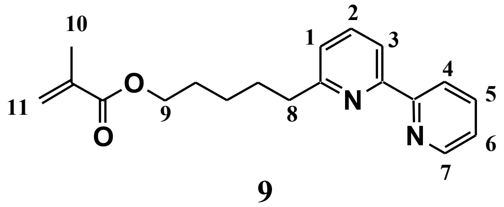


**9**

6-[5-Pentan-1-ol]-2,2'-bipyridine **(8)** (1.57 g, 6.46 mmol) was dissolved in 80 mL of dichloromethane and then, triethylamine (1.96 g, 18.4 mmol) and methacrylic anhydride (3.98 g, 25.8 mmol) were added to the solution, followed by stirring for 2 days at r.t. This procedure was carried out under the Ar atmosphere. The unreacted reagent and salt were removed from the reactant by extraction with saturated $NaHCO_3$ solution and $NH_4Cl$ solution. After dehydration by magnesium sulfate and condensation of the reaction solution, the product solution was further purified by silica column using ethyl acetate/hexane ＝ 1/5 as mobile phase. The obtained solution was condensed and dried in vacuum to recover the product (yields: 1.14 g, 57%)。 $^1$H-NMR(500 MHz, $CDCl_3$) δ: 8.67(1 H, dq, J = 0.9, 4.9, **7**), 8.43(1 H,d, J = 7.9, **3**), 8.19(1 H, dd, J = 0.9, 7.3, **4**), 7.81-7.80(1 H,,m, **2**), 7.71(1 H, t, J = 7.9, **5**), 7.30-7.28(1 H, m, **6)**, 7.15(1 H, d, J = 7.3, **1**), 6.07(1 H, s, **11**), 5.52(1 H, s, **11**), 4.16(2 H, t, J = 6.7, **9**), 2.88(2 H, t, J = 7.3, **8**), 1.92(3 H, s, **10**), 1.90-1.84(2 H, m), 1.79-1.73(2 H, m), 1.51-1.49(2 H, m)

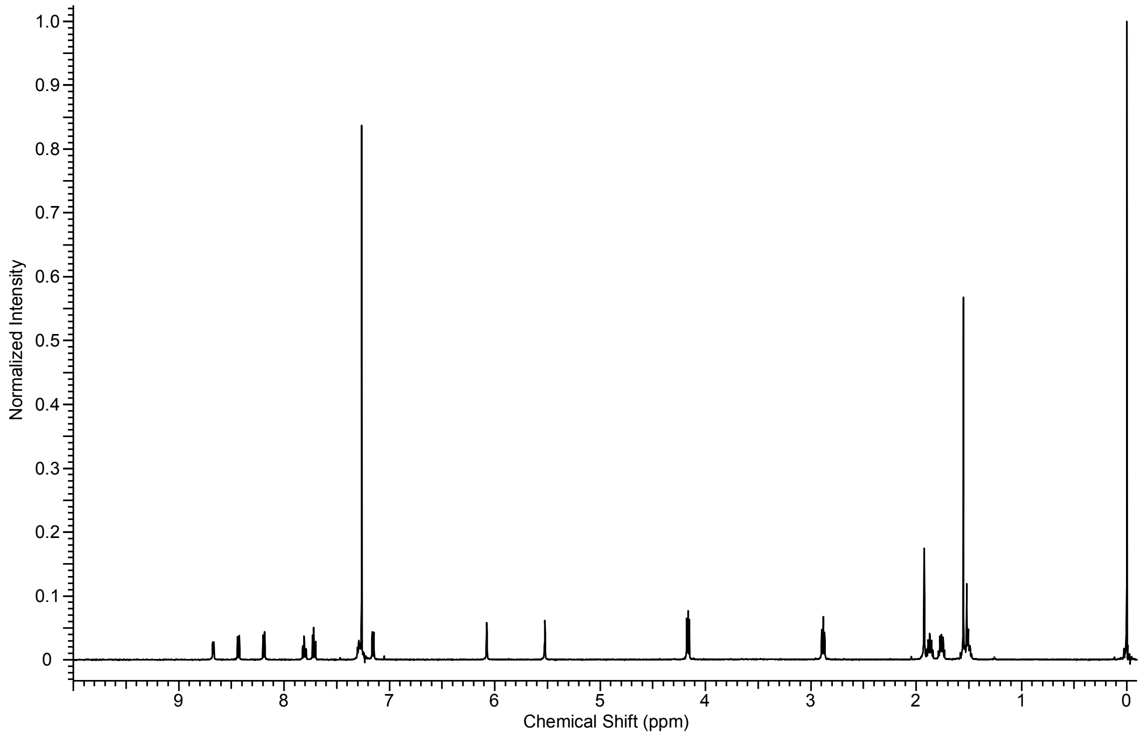


Figure S1. H-NMR spectra of finally obtained product, BPyMA

## SI. 3 Synthesis of p(PEGMA-co-BPyMA-Pt)

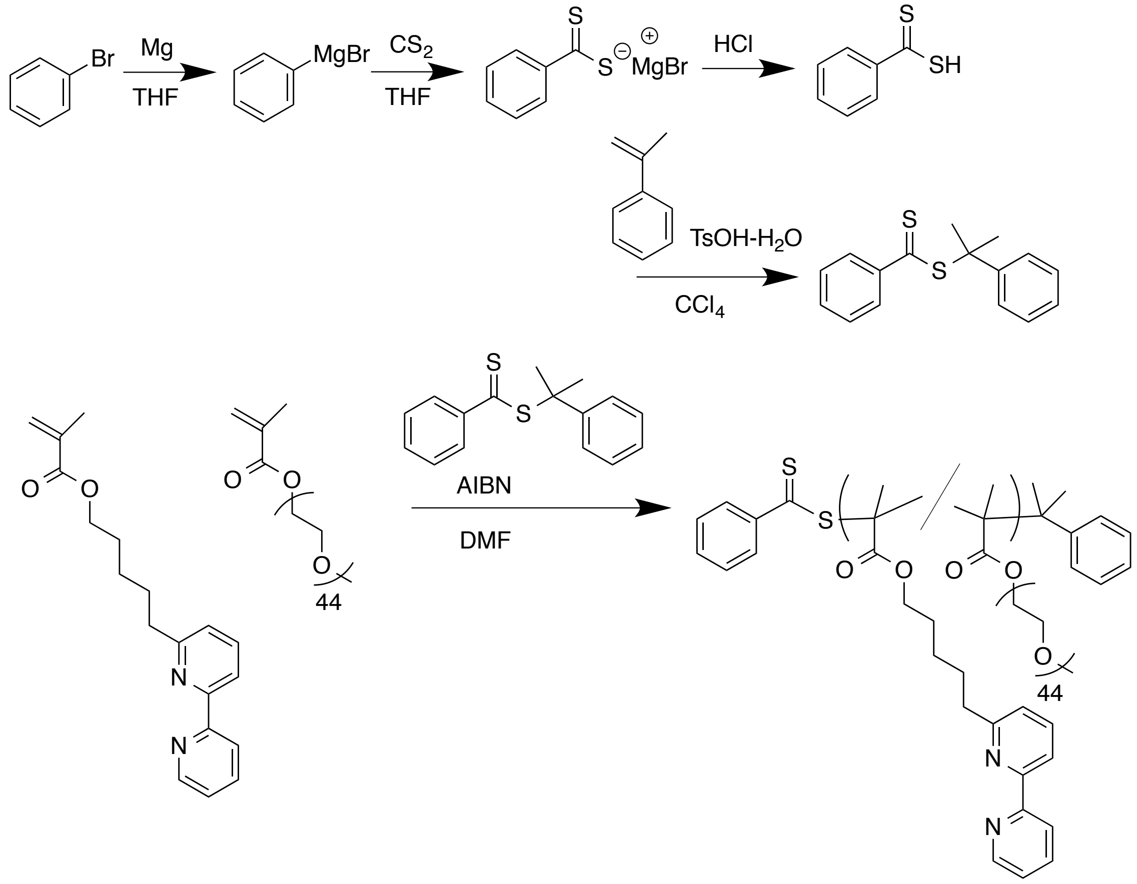


## SI. 3-1 Synthesis of Cumyl dithiobenzoate (CDB) (12)

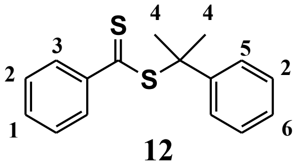


**12**

Magnesium (1.130 g, 46.5 mmol) was put into 60 mL of dehydrated tetrahydrofuran (THF) and refluxed under Ar atmosphere for 10 min to remove passivating magnesium oxide layer on the surface. Bromobenzene **(10)** (7.29 g, 46.5 mmol) was slowly dropped into the magnesium THF solution and stirred for 15 min at 60 ºC. Then, the reaction solution was cooled on the ice and carbon disulfide (3.89 g, 51.1 mmol) was slowly dropped into the reaction solution, followed by stirring for 1.5 hours on ice and another 30 min at r.t. The reaction solution was poured into 1 L of cold water and 1 N HCl was

added to adjust pH to 1.0. The product was collected from the reaction solution by extraction with diethylether three times, condensation and drying in vacuum. Thus obtained product **(11)** (6.41 g, 41.6 mmol) and TsOH-$H_2O$ (158 mg, 0.82 mmol) was dissolved in 100 mL of $CCl_4$ under Ar atmosphere and α-methylstyrene (7.4 g, 62.4 mmol) was slowly added into the solution. The reaction solution was refluxed overnight. Then, reaction solution was condensed by evaporation and dissolved in chloroform, followed by removing the unreacted reagent and salts by extraction using saturated $NaHCO_3$ solution and brine. The obtained chloroform fraction was dehydrated by magnesium sulfate and then, concentrated. The obtained product solution was further purified by silica column using hexane as mobile phase. The product was recovered by drying in vacuum (yields: 1.02 g, 9%). The obtained product was characterized by $^1$H-NMR (JEOLAL-500, 500 MHz, $CDCl_3$) δ: 7.85(2 H, dd, J = 8.5, 1.2 Hz, **3**), 7.55(2 H, d, J = 8.4 Hz, **5**), 7.45(1 H, t, J = 7.5 Hz, **1**), 7.32(4 H, t, J = 7.8 Hz, **2**), 7.22(1 H, t, J = 7.3 Hz, **6**), 2.01(6 H, s, **4**).

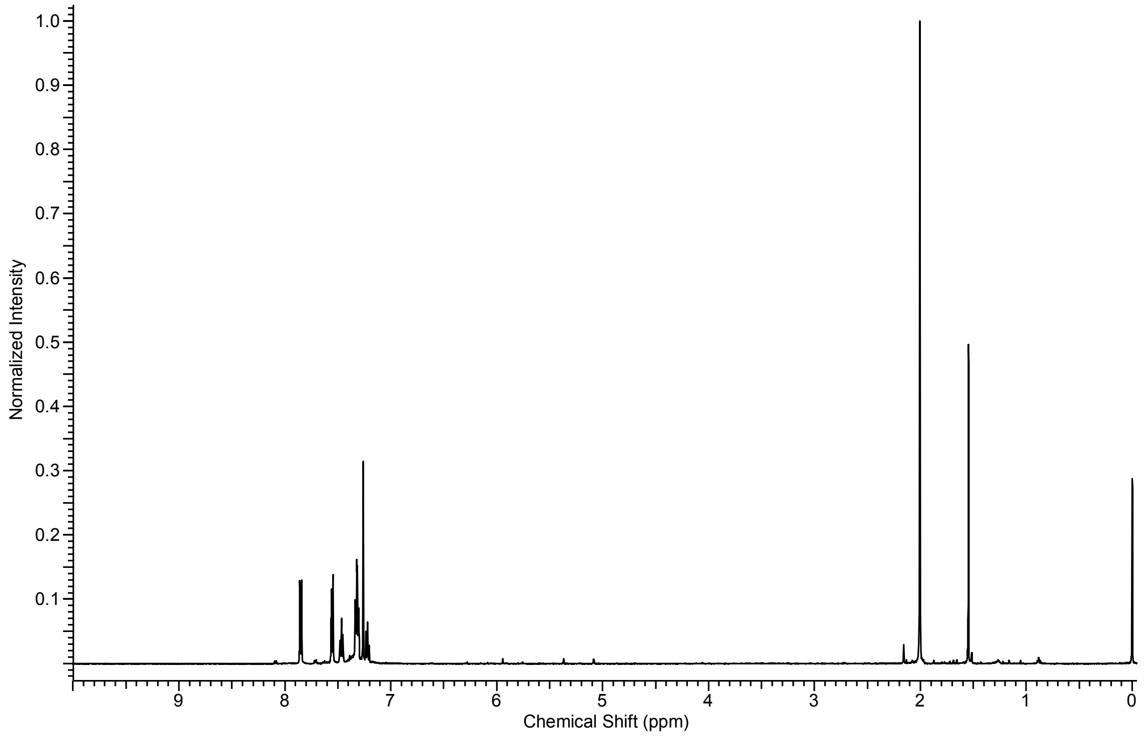


Figure S2 $^1$H-NMR spectra of CDB

**SI. 3-2 Copolymerization of PEGMA and BPyMA monomers**

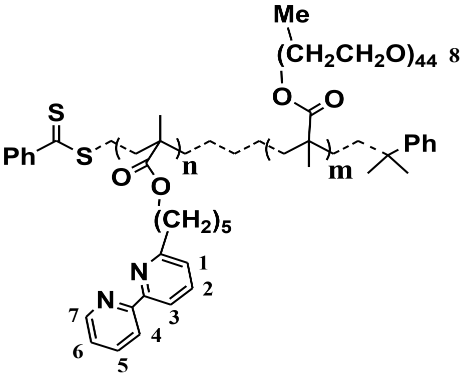


**14-15**

BPyMA **(9)** (714 mg, 2.30 mmol), MeO-PEGMA ($M_n$ = 2089) **(13)** (2.87 g, 1.37 mmol), AIBN (1.39 mg, 8.5 μmol), and CDB (11.5 mg, 42.3 μmol) were dissolved in 20 mL of N, N-dimethylformamide (DMF) as described on Table S1. Freeze-Pump-Thaw cycling was carried out 4 times for removing oxygen from the mixture, followed by stirring at 70ºC for 3 days under Ar atmosphere. The reaction solution was poured into the excess amount of isopropylalchol/diethylether = 1/20 mixture to precipitate the product, followed by benzene freeze-drying to recover the product. The product was characterized by GPC (HLC-8020GPC system, TOHSO, Japan) equipped with TSKgel SuperHZM-H (TOSOH, Tokyo) using THF containing 20 mM triethylamine as an elution, static light scattering measurement (DLS-7000, Otsuka Electronics Co., Osaka, Japan) and $^1$H-NMR (JEOLAL-500 500MHz, $CDCl_3$) δ: 8.66-8.58(**7**), 8.42-8.34(**3**), 8.18-8.08(**4**), 7.80-7.56(**2**, **5**), 7.25-7.20(**6**), 7.16-7.0(**1**), 3.75-3.42(**8**)

Copolymerization of monomer **9** and **13** were carried out via RAFT polymerization using the prepared CDB **12** as a chain transfer agent. Two monomer conversion% were evaluated by comparison between peak intensity derived from methacryrol group proton (6.10-6.06, 5.54-5.50 ppm) and proton on the bipyridine for monomer **9**, and comparison between peak intensity derived from proton on methacryloyl group (6.16-6.12, 5.60-5.56 ppm) and methylene proton of PEG unit, respectively. Completely monomers conversions give 81 kDa in molecular weight under the assumption that CDB well controls RAFT polymerization. The monomer conversion % after 2.5 days

reaction is comparable between monomer **9** and **13** (both 46%) (Figure S3, TableS1). The obtained product after polymerization was calculated to be composed of 27 units of monomer **9** and 14 units of monomer **13**, ratio of which is quite similar to the monomer ratio of polymerization mixture (= 65/35, which was determined from H-NMR spectra of the reactant) for the RAFT polymerization, indicating two monomers consumption rate is comparable. Moreover, SEC analysis showed unimodal peak with 1.29 in molecular weight distribution ($M_w/M_n$) (Figure S5). These results suggest RAFT polymerization proceed in well-controlled manner and two monomers made random copolymer, p(PEGMA-*co*-BPyMA). It should be noted that $M_\mathrm{n}$ of the obtained p(PEGMA-*co*-BPyMA) was 1,1424 Da in PEG standard, quite lower compared to theoretical $M_\mathrm{n}$ calculated based on monomer conversion % obtained by $^1$H-NMR with an assumption that CDB well controlled polymer chain number (Table S1). Probably, the obtained p(PEGMA-*co*-BPyMA) may not have as large dynamic diameter as PEG in DMF. The obtained p(PEGMA-*co*-BPyMA) showed 33520 in apparent molecular weight by static light scattering measurement (Table S1, Figure S6).

Table S1. Summary of obtained p(PEGMA-*co*-BPyMA)

| Conversion % | | Monomer **9**/Monomer **13** | | | |
|---|---|---|---|---|---|
| Monomer **9** | Monomer **13** | Polymerization mixture | Obtained structure | $M_{\text{n-theoretical}}$ [(a)] | $M_{\text{app}}$ [(b)] |
| 46 | 46 | 65/35 | 27/14 | 38900 | 33520 |

[(a)]Predicted from monomer conversion obtained by $^1$H-NMR
[(b)]Obtained by SLS measurement

(a)

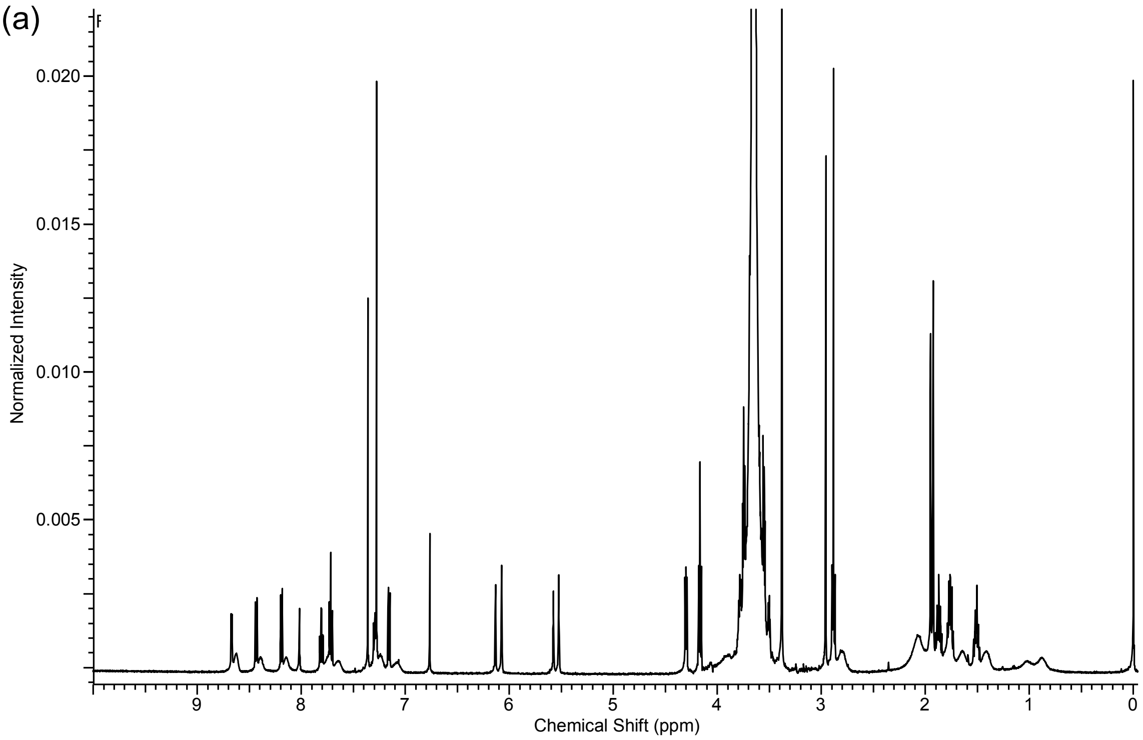


(b)

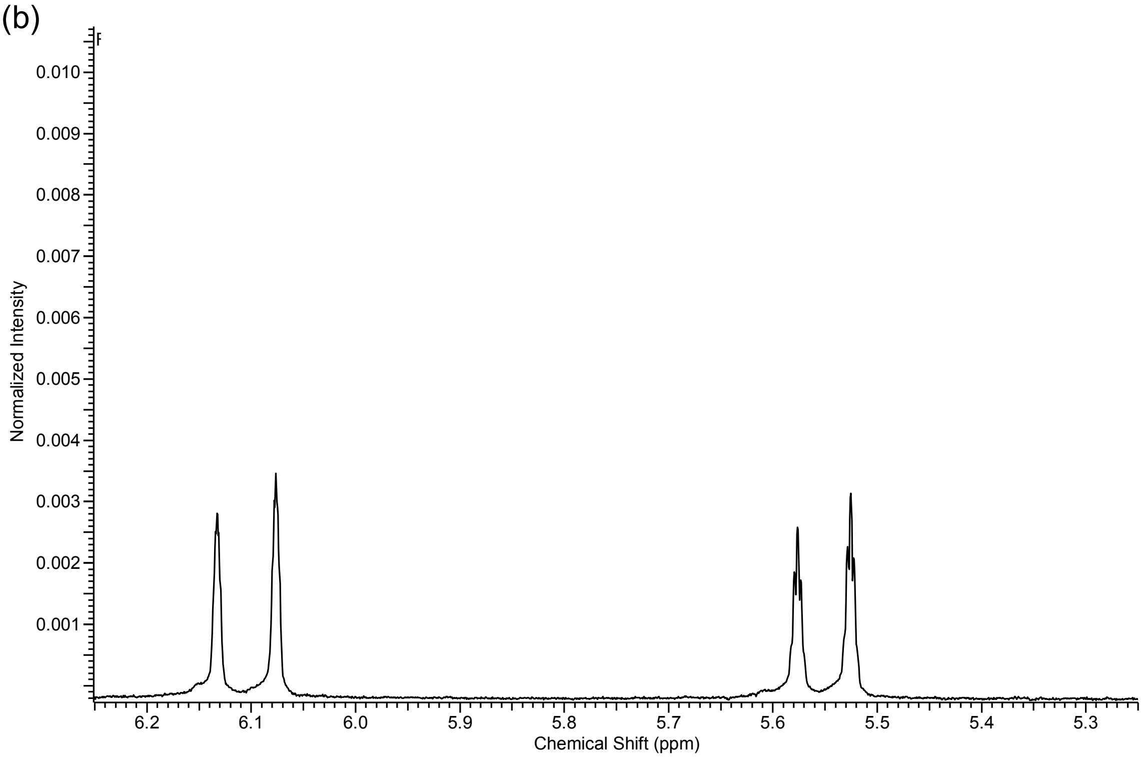


Figure S3. $^{1}$H-NMR spectra of the dried up polymerization reactant to predict monomer conversion % in the reactant (a) and magnified image from 5.25 to 6.25 ppm (b).

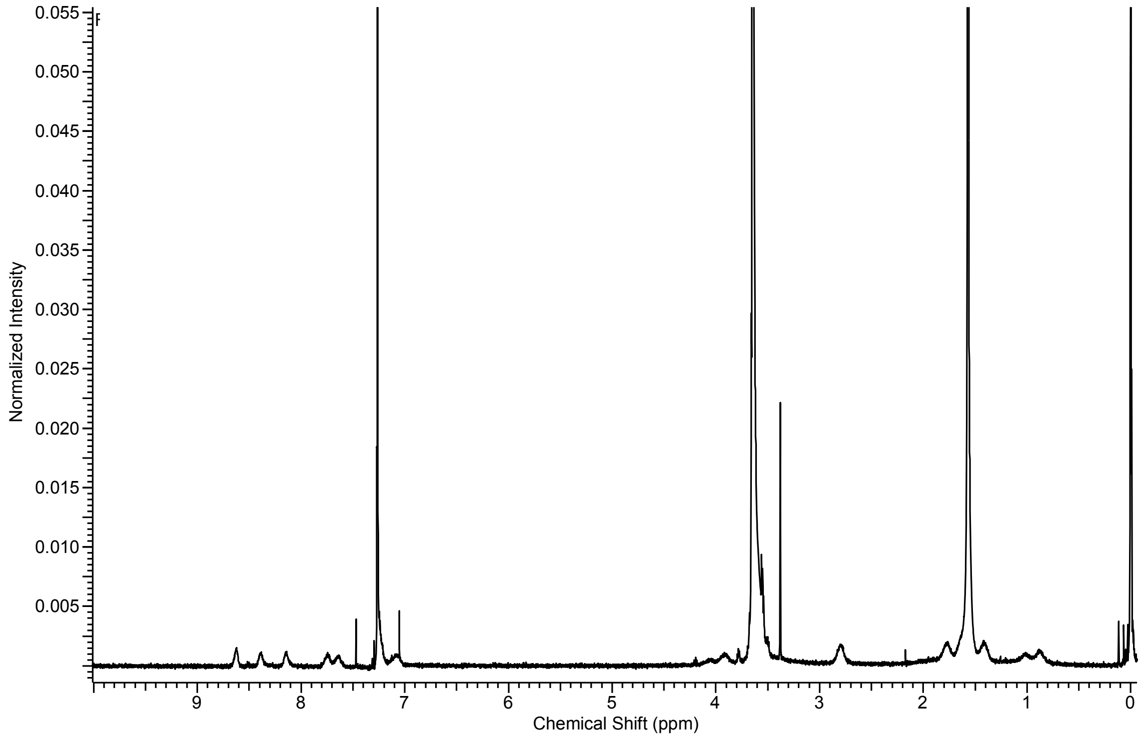


Figure S4. $^{1}$H-NMR spectra of the obtained p(PEGMA-*co*-BPyMA)

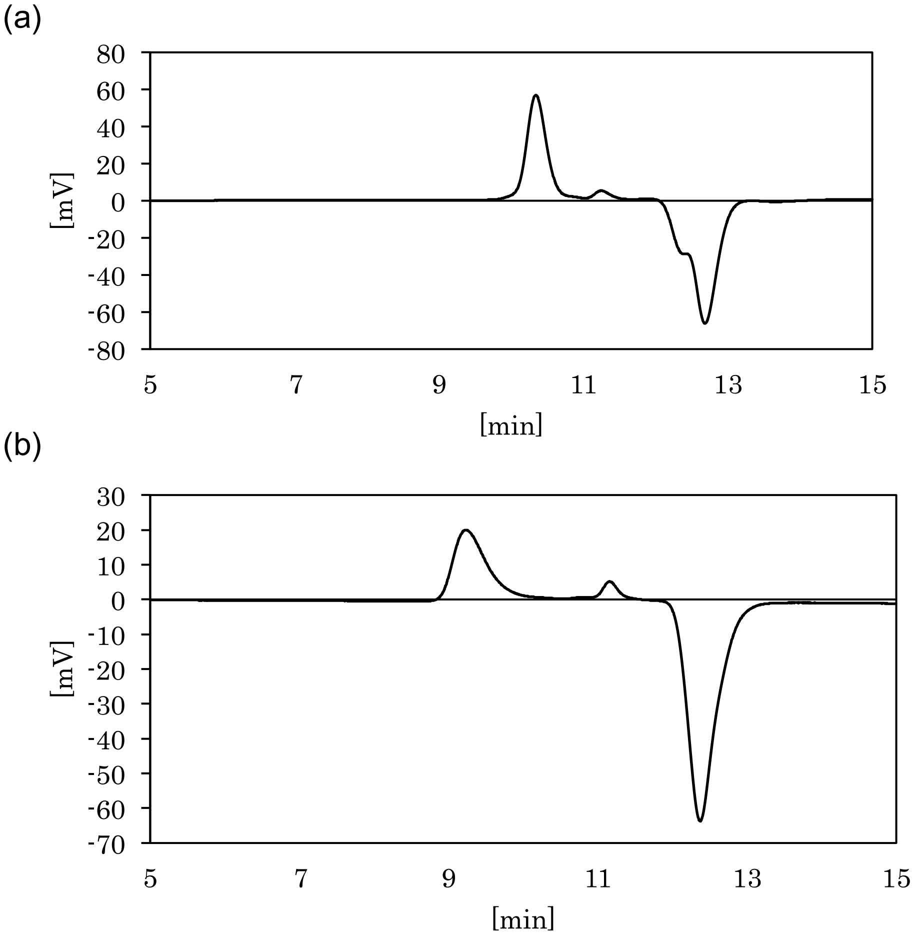


Figure S5. GPC curves of PEGMA monomer (a) and p(PEGMA-*co*-BPyMA) (b)

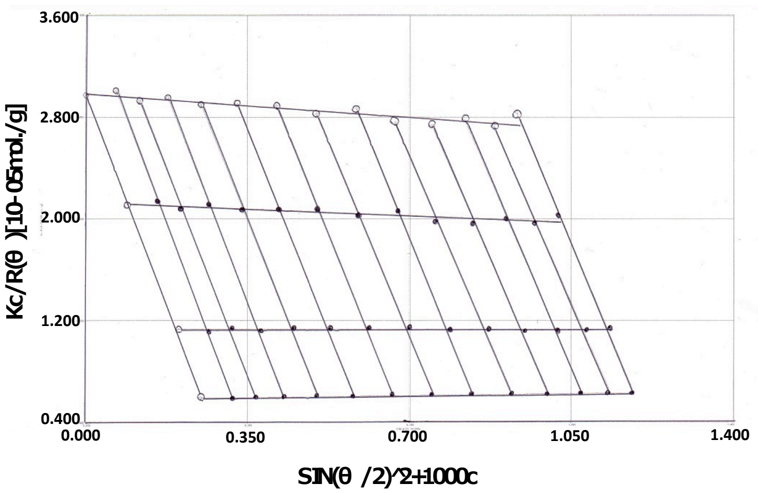


Figure S6. Zimm plot of p(PEGMA-*co*-PByMA)

## SI. 4 Formation of metal complex with BPy unit

### SI. 4-1 Preparation of [Pt(DMSO)Cl₂](DMSO-Pt)

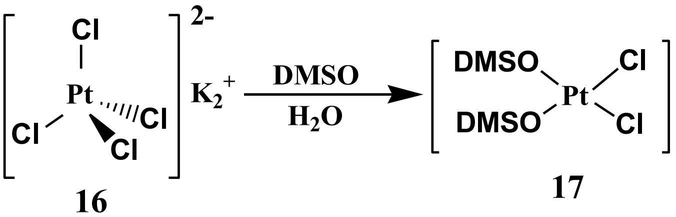


16 17

To smoothly form metal complex of 2,2'-bipyridine group and Pt, [Pt(DMSO)$Cl_2$](DMSO-Pt) was synthesized as a reaction intermediate compound. Potassium tetrachloroplatinate(Ⅱ) (300 mg, 723 μmol) was dissolved in 1.5 mL of deionized water. DMSO (154 μL, 2.17 mmol) was slowly added into the solution on ice, followed by stirring on ice for two hours. Then, precipitate was collected by filtration. The obtained product was washed by ethanol and diethyl ether on the filter, followed by drying in vacuum to recover product as a white powder (yields: 248 mg, 81%). The obtained product was characterized by FT-IR spectrum measurement (Nicolet 6700 FT-IR, Thermo Fisher Sci. Co., Kanagawa, Japan) using the ATR methods. The measurement was performed from 650 $cm^{-1}$ to 4000 $cm^{-1}$ in wavenumber range and 100 scans accumulation was processed to data. The obtained product was further characterized by elemental analysis (2400 II CHNS/O, PerkinElmer Japan, Yokohama, Japan) as follows: Two mg of the obtained product was put on a tin thin film, and then the tin films were processed into cubes to analysis the elements (C, H, and O).

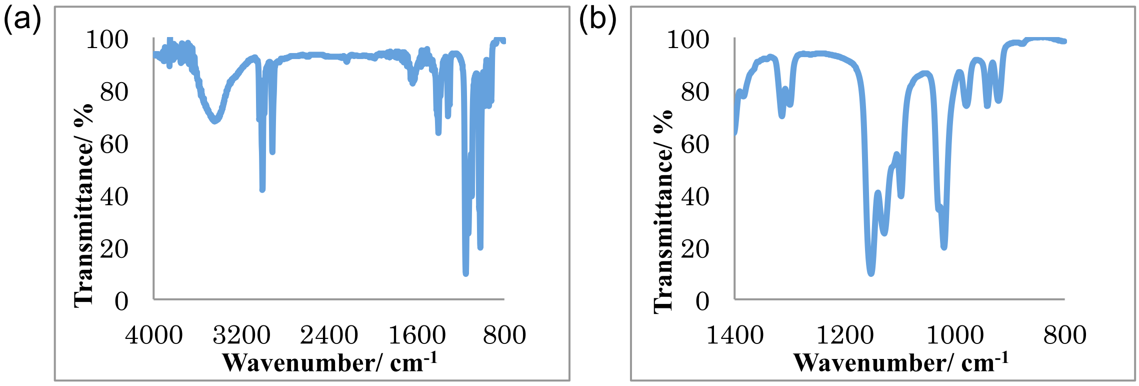


Figure S7. FT-IR spectrum of DMSO-Pt (a) and the magnified image from 800 to 1400 $cm^{-1}$.

Table S2. Elemental analysis of the obtained DMSO-Pt **(17)**

| | Theoretical value (%) | | | Measured value (%) | | |
|---|---|---|---|---|---|---|
| | C | H | N | C | H | N |
| DMSO | 30.7 | 7.7 | 0 | - | - | - |
| DMSO-Pt **(17)** | 11.4 | 2.8 | 0 | 10.8 | 2.50 | 0.03 |

FT-IR spectrometry of the DMSO-treated potassium tetrachloroplatinate(Ⅱ) showed peak at 1128 and 1152, which may be peak of sulfoxide group (S=O) vibration shifted from an original peak of sulfoxide group (S=O) of DMSO at 1057 $cm^{-1}$ by forming coordinate bond with Pt and changing vibration of S=O (Figure S7). The obtained peaks were quite similar to previously reported peaks of sulfoxide group of DMSO-Pt (1128, 1152 $cm^{-1}$) [M. V. Babak, et. al, *Inorg. Chem.* 2018, **57**, 2851., J. H. Price, et. al, *Inorg. Chem.*, 1972, **11**, 1280.]. In addition, elemental analysis of the obtained DMSO-treated potassium tetrachloroplatinate (II) showed almost the same value as theoretical DMSO-Pt (Table S2), further supporting successful formation of metal complex of DMSO-Pt.

**SI. 4-2 Formation of Pt complex with BPy**

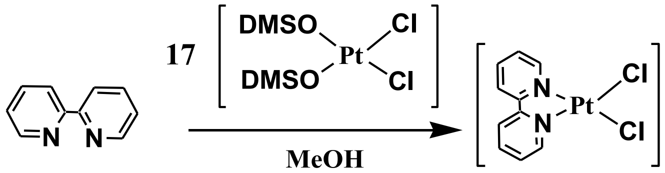


Pt complex with BPy (BPy-Pt) was prepared as a control of monovalent DNA

intercalator. DMSO-Pt (60 mg, 0.14mmol) and 2'2-Bipyridine (22 mg, 0.14mmol) were dissolved in 100 mL of MeOH and stirred for 24 hours at r.t. The precipitate was filtered to collect, followed by washing using diethyl ether and $CHCl_3$, and drying in vacuum to recover product as yellow powder (yields: 44 mg, 74%). The obtained product was characterized by $^1$H-NMR (JEOLAL-500, 500MHz, $CDCl_3$), FT-IR spectrum measurement (Nicolet 6700 FT-IR, Thermo Fisher Sci. Co., Kanagawa, Japan) using the ATR methods, and elemental analysis (2400 II CHNS/O, PerkinElmer Japan, Yokohama, Japan) as follows: Two mg of the obtained product was put on a tin thin film, and then the tin films were processed into cubes to analysis the elements (C, H, and O).

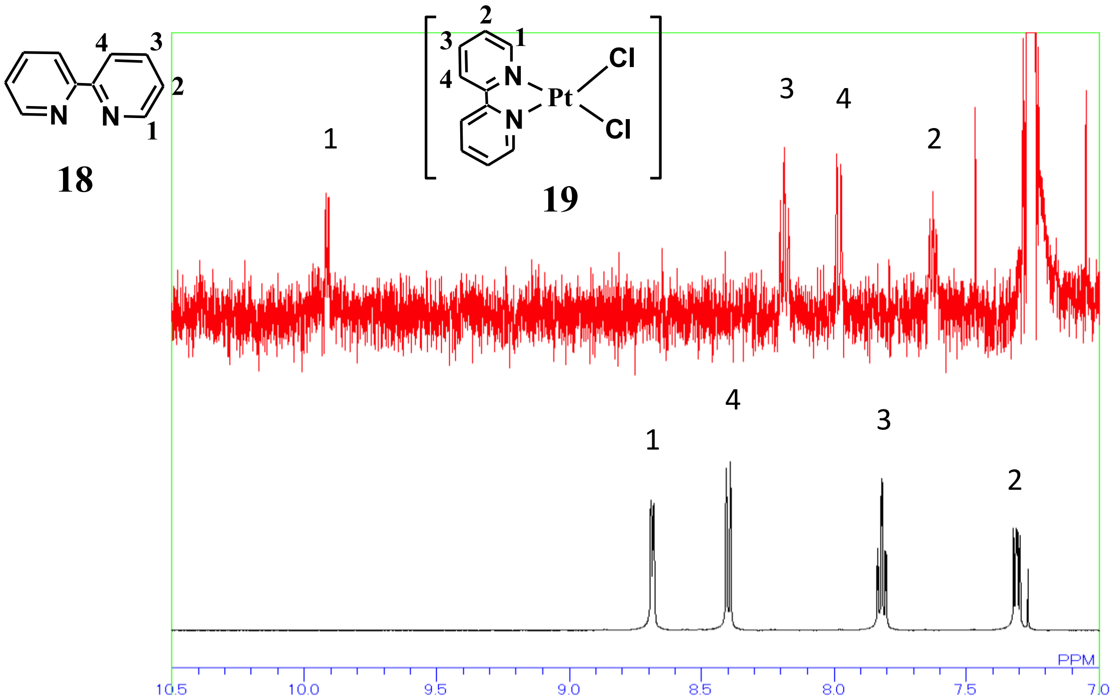


Figure S8 $^1$H-NMR spectra of BPy after (above) and before (bottom) complexiation with Pt.

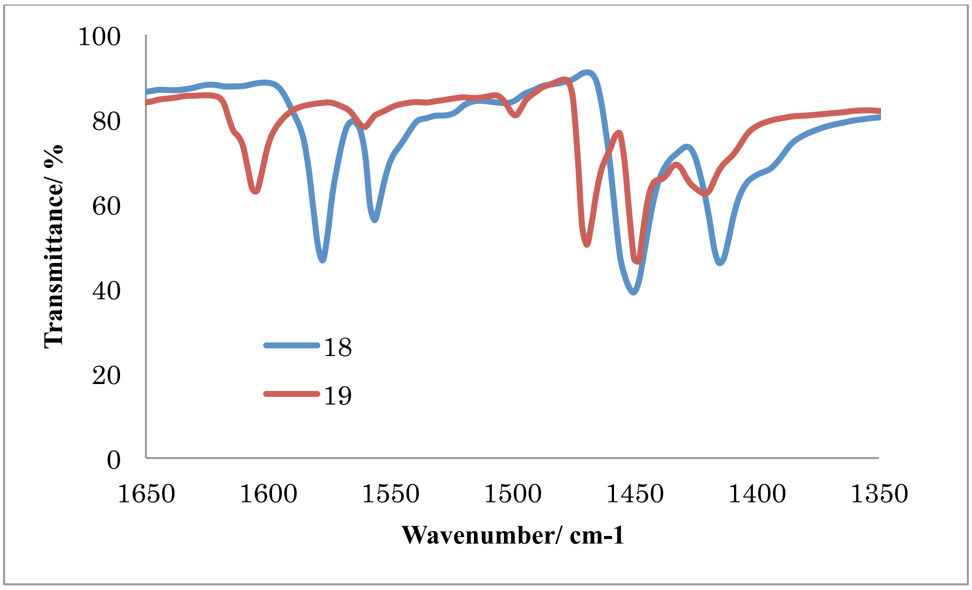


Figure S9. FT-IR spectra of 2,2'-Bipyridine (blue line) and BPy-Pt (red line) from 1350 to 1650cm$^{-1}$

Table S3. Peak derived from C=C and C=N vibration

| 2,2'-Bipyridine **(18)** | BPy-Pt **(19)** |
|---|---|
| 1415 cm$^{-1}$ | 1448 cm$^{-1}$ |
| 1450 cm$^{-1}$ | 1469 cm$^{-1}$ |
| 1556 cm$^{-1}$ | 1560 cm$^{-1}$ |
| 1577 cm$^{-1}$ | 1604 cm$^{-1}$ |

Table S4. Elemental analysis of BPy-Pt

| | Theoretical value (%) | | | Measured value (%) | | |
|---|---|---|---|---|---|---|
| | C | H | N | C | H | N |
| 2,2'-Bipyridine | 76.9 | 5.1 | 17.9 | - | - | - |
| BPy-Pt | 28.4 | 1.9 | 6.63 | 27.4 | 1.53 | 6.30 |

In comparison between the obtained sample and 2,2'-bipyridine, peak derived from protons on 6-position and 6'-position of bipyridine was shifted to higher ppm region in $^{1}$H-NMR spectra (Figure S8) probably because of forming coordinal bonds with Pt. In FT-IR spectra measurement, peaks derived from vibration of C=C and C=N bonds were shifted to shorter wavelength region (Figure S9, Table S3), also supporting formation of the Pt complex [J. H. Price, et. al, *Inorg. Chem.*, 1972, **11**, 1280.]. The elemental analysis of the obtained sample showed C, H, and N weight were slightly smaller value

from theoretical value (Table S4) as well as C and H in DMSO-Pt (Table S2). This is probably because sample contained a little amount of the by-product without C, H, and N, potentially potassium chloride.

## SI.4-3 Preparation of Pt complex with p(PEGMA-*co*-BPyMA)

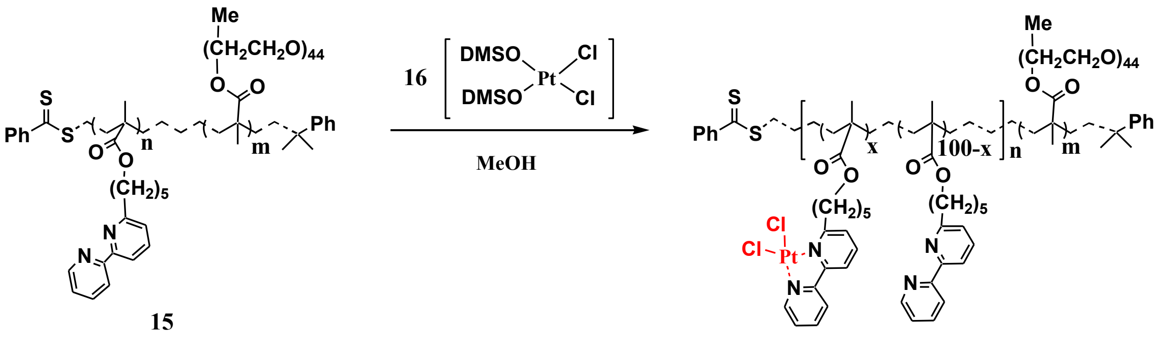


Scheme S4. Preparation of Pt complex with P(PEGMA-*co*-BPyMA)

p(PEGMA-*co*-BPyMA) and DMSO-Pt were dissolved in MeOH and stirred at r.t. for 24 hours. The product was recovered by drying in vacuum after dialysis against MeOH 3 times. The feed ratios for the reaction were listed on Table S5 and reaction solvent, MeOH amounts were 40 mL for 50/50 and 100/0 batches, and 8 mL for 23/77 and 67/33 batches. The obtained product was characterized by $^1$H-NMR (JEOLAL-500, 500MHz, $CDCl_3$).

Table S5. Feed ratios for preparation of p(PEGMA-*co*-BPyMA) having various Pt

| | Polymer / mg | BPy molar / μmol | DMSO-Pt / μmol | DMSO-Pt /BPy molar ratio |
|---|---|---|---|---|
| 23/77 | 40 | 28 | 6.3 | 0.23 eq |
| 50/50 | 200 | 140 | 70 | 0.5 eq |
| 67/33 | 40 | 28 | 18.8 | 0.67 eq |
| 100/0 | 200 | 140 | 140 | 1.0 eq |

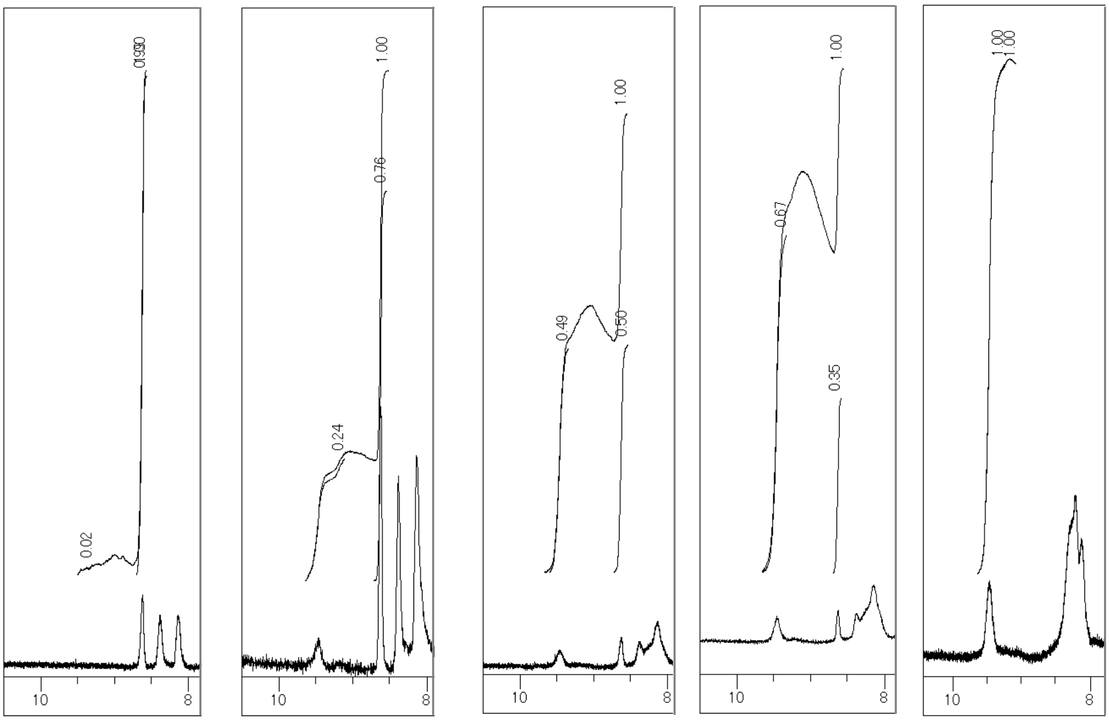


Figure S10 $^{1}$H-NMR spectra of (a) original P(PEGMA-*co*-BPyMA), and Pt complex with P(PEGMA-*co*-BPyMA) of feed ratios Pt/BPy = (b)23/77, (c)50/50, (d)67/33, (e)100/0.

Table S6. Summary of $^{1}$H-NMR measurement for series of Pt complex with p(PEGMA-*co*-BPyMA-Pt)

| Feed molar ratio of Pt/BPy | 9.5 ppm | 8.5 ppm |
|---|---|---|
| 0/0 | 0.02 | 0.99 |
| 23/77 | 0.24 | 0.76 |
| 50/50 | 0.49 | 0.50 |
| 67/33 | 0.67 | 0.35 |
| 100/0 | 1.00 | 0 |

Formation of BPy-Pt complex shifted peak derived from protons on 6-position and 6'-position of bipyridine from the original ones in $^{1}$H-NMR spectra as described in SI. 4-2. Thus, BPy-Pt/BPy complex ratios in p(PEGMA-co-BPyMA) were calculated by comparison between peak intensity derived from shifted (δ9.5 ppm) and original (δ8.5 ppm) 6-position and 6'-position of bipyridine signal (Figure S10). The obtained values are all consistent with feed ratios (Table S6), suggesting successful preparation of Pt

complexes with p(PEGMA-*co*-BPyMA) having various BPy-Pt/BPy ratios.

**SI.5 Evaluation on binding constant of Ethidium Bromide (EtBr) to DNA.**

CT-DNA was dissolved in PBS containing 1.5% DMSO and the CT-DNA solutions were adjusted to 0.3 and 0.035 mg/mL of CT-DNA concentration, and 2480 μL in volume using Jasco V-650 spectrometer (JASCO Co., Tokyo, Japan). Then, 2 μL of 1 mg/mL EtBr solution was added to the CT-DNA solution and fluorescence at 580 nm exited by 510 nm were measured with Jasco FP-6500 (JASCO Co., Tokyo, Japan) after 10 min incubation. Another 2 μL of the EtBr solution were further added to the measured solution and then, analyzed in same manner as the above. This procedure was repeated up to reaching total EtBr solution addition to 20 μL.

(a) (b)

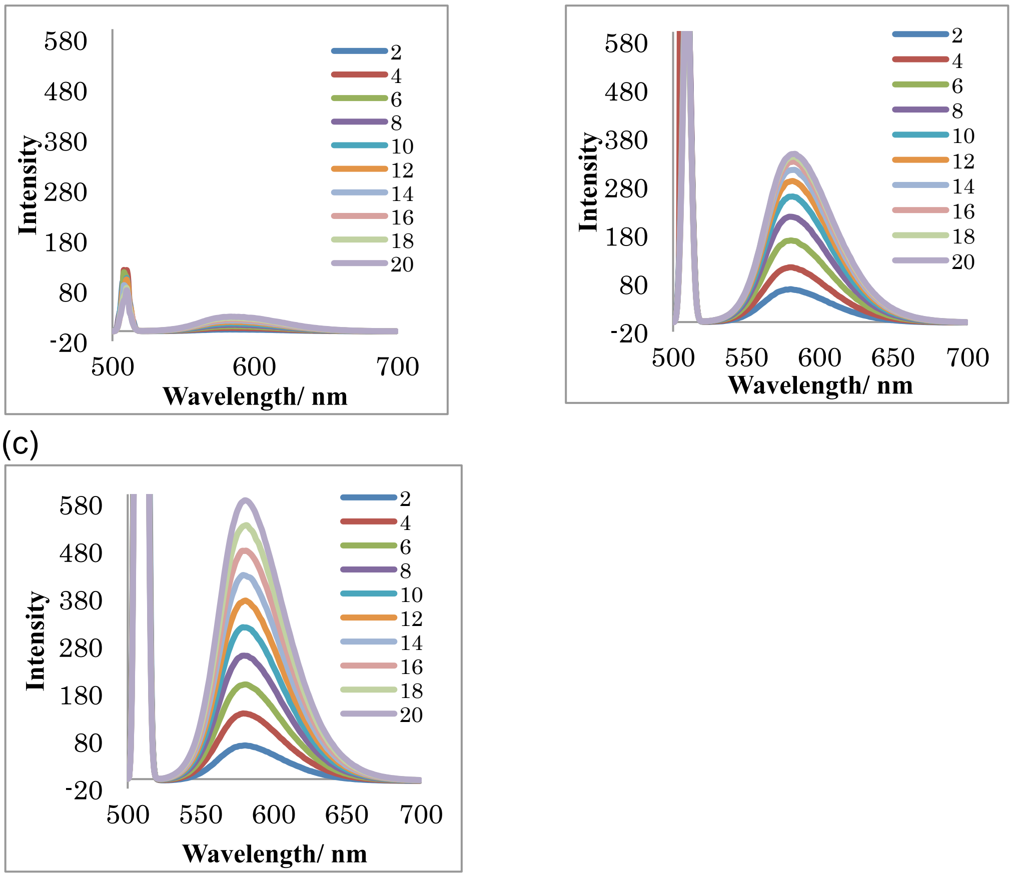


(c)

Figure S11. Fluorescence spectra of (a) 0, (b) 0.035 and (c) 0.3 mg/mL of CT-DNA solution with escalating EtBr amounts exited by 510 nm.

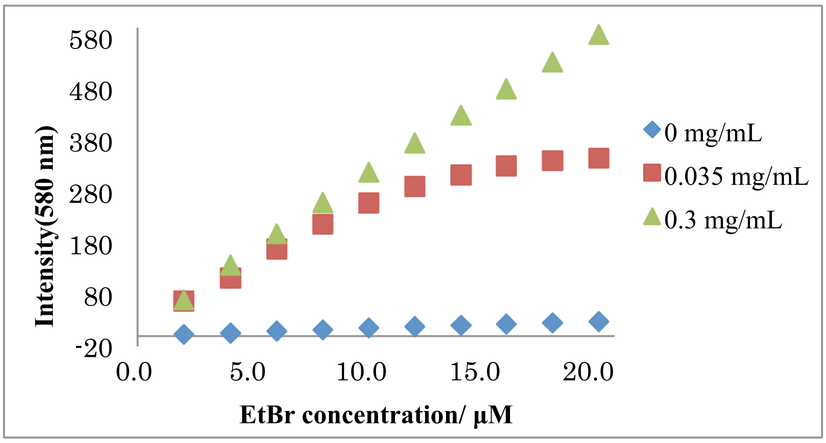


Figure S12. Fluorescence intensity at 580 nm of 0 (a), 0.035 (b) and 0.3 (c) mg/mL of CT-DNA solution with escalating EtBr amounts.

When the concentration of EtBr increase, emission at 580 nm increase slightly as shown in Figure S11(a) because the EtBr itself has some degree of fluorescence without binding to DNA. As for 0.3 mg/mL of CT-DNA solution, fluorescence intensity linearly increased by escalating EtBr concentration (Figure S11(c)), indicating all of the added EtBr bound to CT-DNA. In contrast, the fluorescence intensity of 0.035 mg/mL of CT-DNA solution increased linearly up to adding 6 μL of the EtBr solution, but the increase ratio become less and less adding above 6 μL and finally seemed to be plateau. This indicated that there are free EtBr molecules not bound to the DNA by adding more than 8 μL of EtBr solution. Binding constant of EtBr to CT-DNA was calculated by drawing Scatchard plot from these fluorescence results of 0, 0.035 and 0.3 mg/mL of CT DNA solutions.

Scatchard plots are on the following equation.

$$\frac{\gamma}{[C_f]} = nK - \gamma K$$

$\gamma$: Moles of EtBr bound to the base per mole in CT-DNA

$[C_f]$: Concentration of free EtBr (not bound to CT-DNA)

$K$: binding constant

$n$: Binding number capacity of EtBr to base per mole in CT-DNA

$\gamma$ can be obtained by dividing $C_b$ (total EtBr number bound to DNA) by total base number containing in CT-DNA solution. Considering the 0.035 mg/mL of CT-DNA

solution, $C_b$ can be calculated by the following equation under the assumption that almost all EtBr were bound to DNA in 0.3 mg/mL of CT-DNA solution because CT-DNA amount is enough large.

$$C_b = C_t \frac{I - I_0}{I_{max} - I_0}$$

$C_t$: Adding amount of EtBr

$I$: Fluorescence intensity obtained from 0.035 mg/mL of CT-DNA solution

$I_{max}$: Fluorescence intensity obtained from 0.3 mg/mL of CT-DNA solution

$I_0$: Fluorescence intensity obtained from 0 mg/mL of CT-DNA solution (fluorescence intensity of original EtBr)

It should be noted that free EtBr amount, $C_f$, can be calculated by subtracting $C_b$ from $C_t$. Using the above equations, the values requiring for Scatchard plots were obtained as described in Table S7.

Table S7. Parameters for Scatchard plots.

| [EtBr]/ M × $10^{-6}$ | $C_b$ / M × $10^{-6}$ | $C_f$/ M × $10^{-6}$ | γ | γ/$C_f$ / $M^{-1}$ |
|---|---|---|---|---|
| 2 | 1.954 | 0.090 | 0.017 | 192469 |
| 4 | 3.313 | 0.771 | 0.029 | 37966 |
| 6 | 5.136 | 0.985 | 0.045 | 46057 |
| 8 | 6.762 | 1.393 | 0.060 | 42882 |
| 10 | 8.171 | 2.014 | 0.072 | 35822 |
| 12 | 9.315 | 2.898 | 0.082 | 28389 |
| 14 | 10.218 | 4.018 | 0.090 | 22460 |
| 16 | 10.930 | 5.327 | 0.097 | 18120 |
| 18 | 11.335 | 6.939 | 0.100 | 14426 |
| 20 | 11.541 | 8.747 | 0.102 | 11652 |

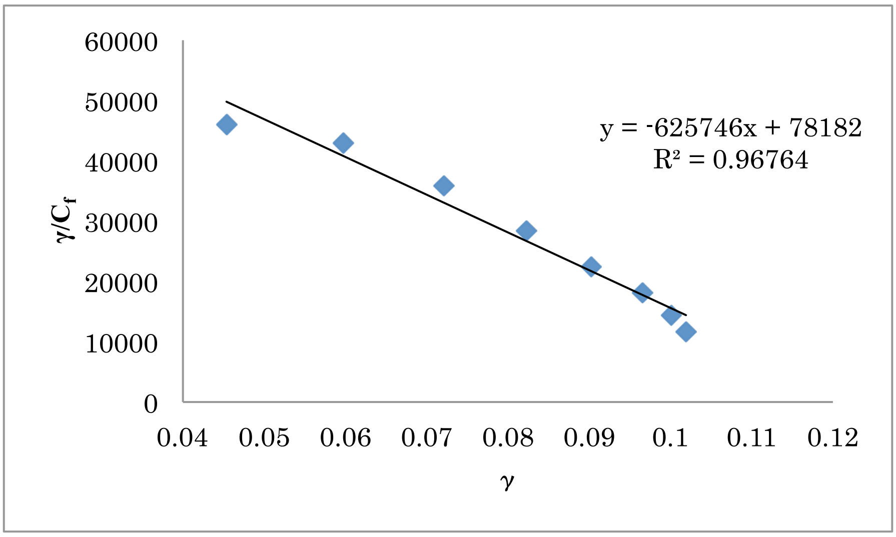


Figure S12. Scatchard plots of EtBr binding to CT-DNA.

From the Scatchard plot, $K$ and $n$ were obtained to be $6.26 \times 10^5$ M and 0.125, respectively. These values are well consistent to the previously reported values [J. B. LePecq, C. Paoletti, *J. Mol. Biol* **1967**, *27*, 87-106].

**SI. 6 Investigation on suitable EtBr/CT-DNA composition of solution for EtBr exclusion assay**

Solutions with CT-DNA concentrations of 0.0025, 0.005, 0.01, 0.015, 0.02 mg/mL were prepared using PBS containing 1.5% DMSO and 4 μM EtBr. Fluorescence intensities of the solutions at 580 nm with excitation of 510 nm were measured at 10 min after preparation using Jasco FP-6500 (JASCO Co., Tokyo, Japan).

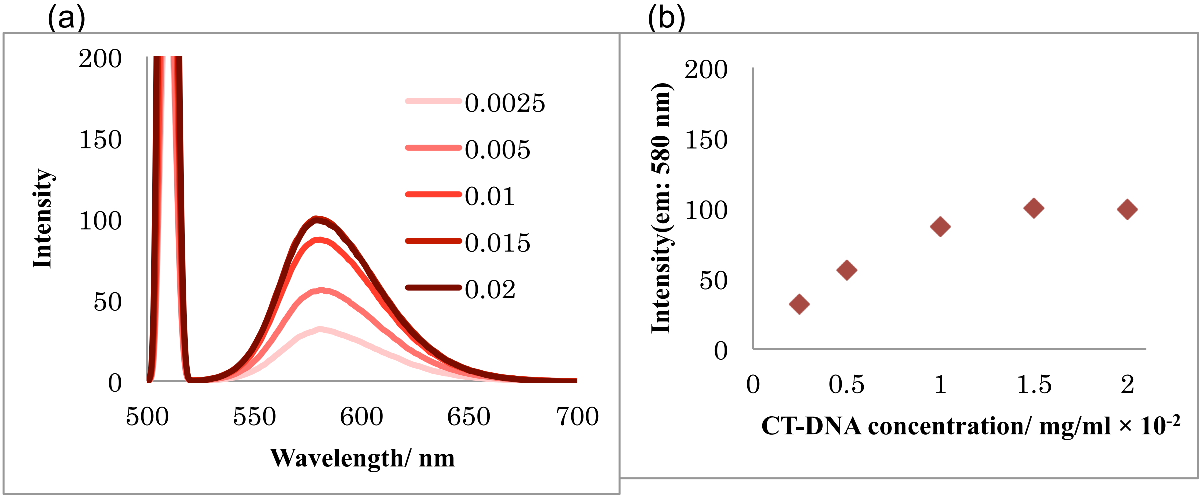


Figure S13. (a) Fluorescence spectra and (b) fluorescence intensity at 580 nm of solutions containing 4 μM EtBr and CT-DNA of 0.0025, 0.005, 0.01, 0.015 and 0.02 mg/mL.

Fluorescence intensity seemed to increase linearly against DNA concentration up to 0.01 mg/mL of DNA concentration and seemed to be plateau between 1.5 and 2.0 mg/mL (Figure S13(b)). DNA concentration for binding without excess or deficiency against 4 μM EtBr was obtained to be 0.0117 mg/mL from the intersection of the extrapolated line fitting on fluorescence linearly increasing region and plateau region in Figure S13(b).

**SI. 7 EtBr exclusion assay for calculation of binding constant of BPy-Pt, p(PEGMA-*co*-BPyMA-Pt), BPy and p(PEGMA-*co*-BPyMA)**

EtBr exclusion assays were performed as described in the main text.

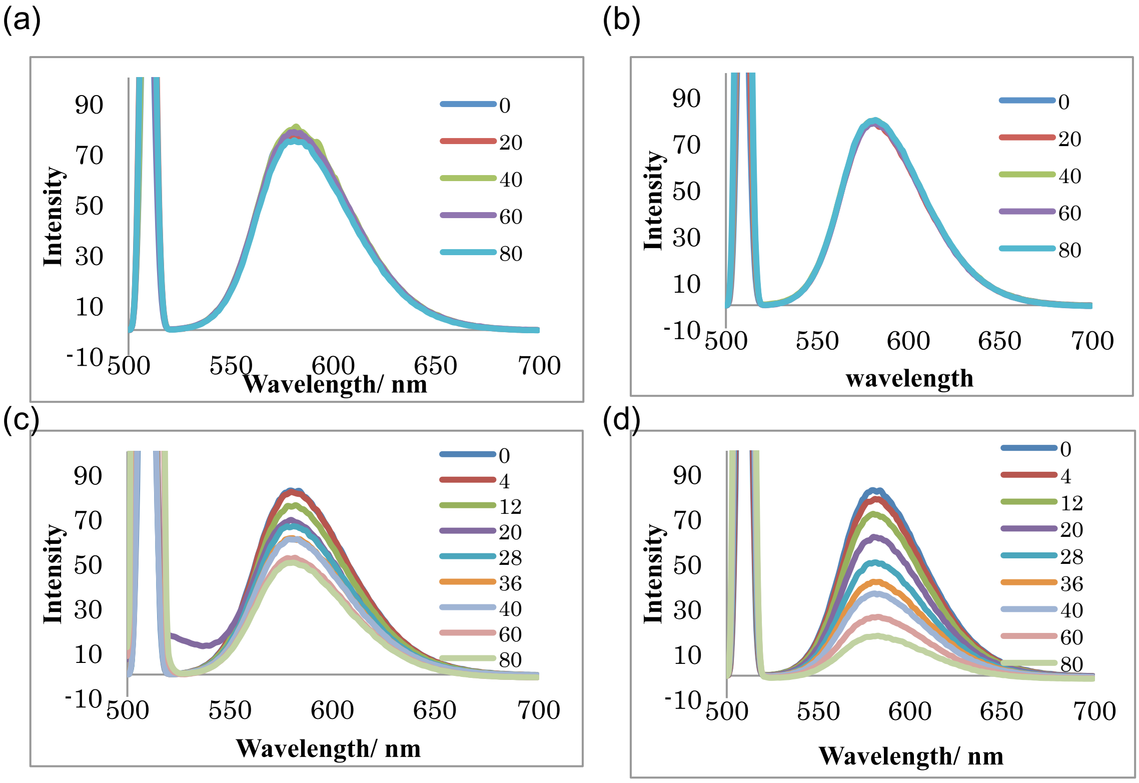


Figure S14. Fluorescence spectra of EtBr and CT-DNA solutions incubated with various concentrations of (a) BPy, (b) p(PEGMA-*co*-BPyMA), (c) BPy-Pt and (d) p(PEGMA-*co*-BPyMA-Pt). The final concentration of BPy or BPy-Pt in the mixtures were varied from 0 to 80 μM.

As for BPy-Pt, EtBr exclusion assay was also performed using PBS containing 2 μM of EtBr and 0.00585 mg/mL of CT-DNA, which is the twice diluted EtBr and DNA solution as the above.

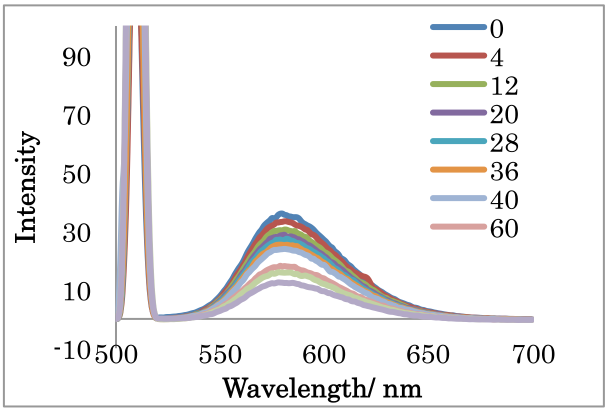


Figure S15. Fluorescence spectra of solutions containing 2 μM of EtBr and 0.00585 mg/mL of CT-DNA.

**SI. 8 Evaluation on structural change of DNA incubated with BPy and p(PEGMA-*co*-BPyMA) by Circular Dichroism (CD) spectrometer**

The CD spectra of DNA incubated with BPy and p(PEGMA-*co*-BPyMA) were obtained in the same manner as described in the main text except using sample solution with BPy concentration of 10 mM.

Table S8. The solutions added to the CT-DNA PBS solutions.

| BPy or p(PEGMA-*co*-BPyMA) solutions (μL) | DMSO (μL) | Conc. of BPy units (μM) | BPy/base molar ratio |
|---|---|---|---|
| 0 | 150.0 | 0 | 0 |
| 28.3 | 121.7 | 28.3 | 0.125 |
| 58.9 | 91.1 | 58.9 | 0.26 |

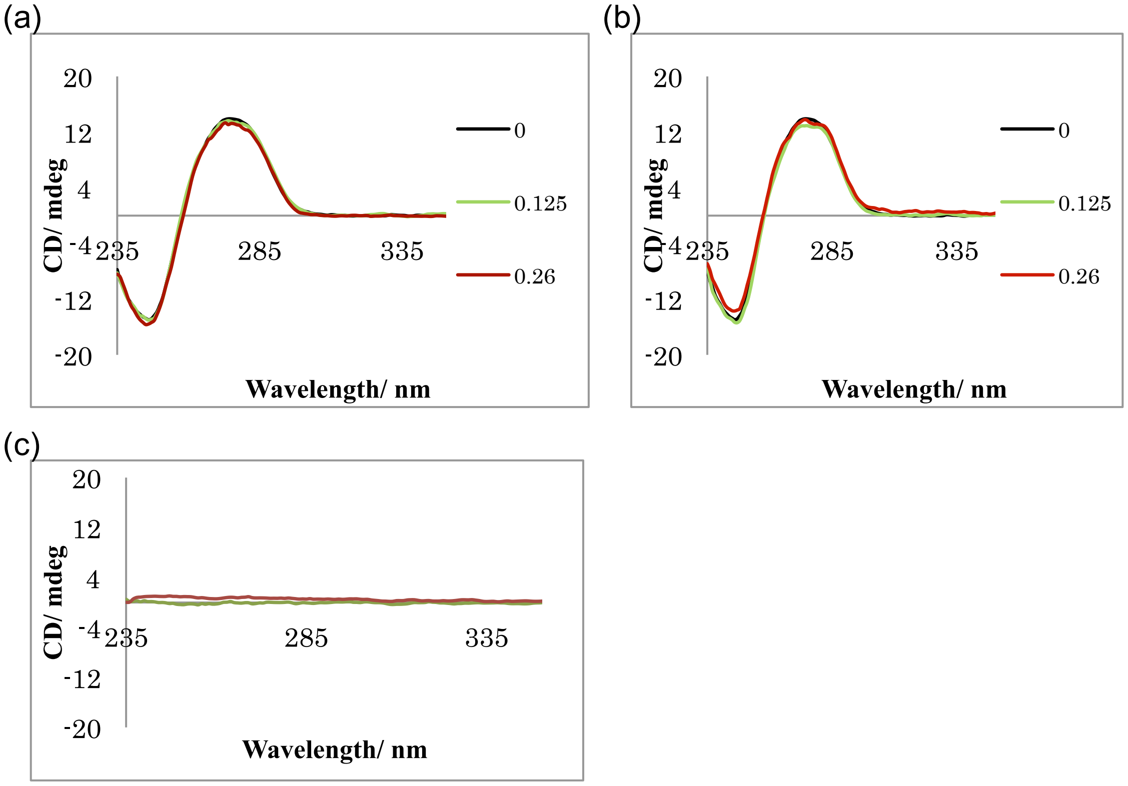


Figure S16. CD spectrum measurements of CT-DNA solutions incubated with (a) BPy and (b) p(PEGMA-*co*-ByMA) at molar ratio BPy/base at 0.125 and 0.26. (c) CD spectrum measurements for 58.9 μM of BPy-Pt and p(PEGMA-*co*-BPyMA-Pt) solutions.

BPy-Pt and p(PEGMA-*co*-BPyMA-Pt) changed the spectrum of CT-DNA as shown in Figure 2 in main text. In contrast, BPy and p(PEGMA-*co*-BPyMA) did not change the spectrum (Figure S16a, b). It should be noted that BPy-Pt and p(PEGMA-*co*-BPyMA-Pt) did not show any signals in CD spectrum measurement. These results suggest changing CT-DNA spectrum requires formation of BPy-Pt complex and probably BPy-Pt planar structure can intercalate to the CT-DNA.

**SI. 9 Observation of pDNA incubated with BPy-Pt and p(PEGMA-*co*-BPyMA-Pt) by atomic force microscopy imaging**

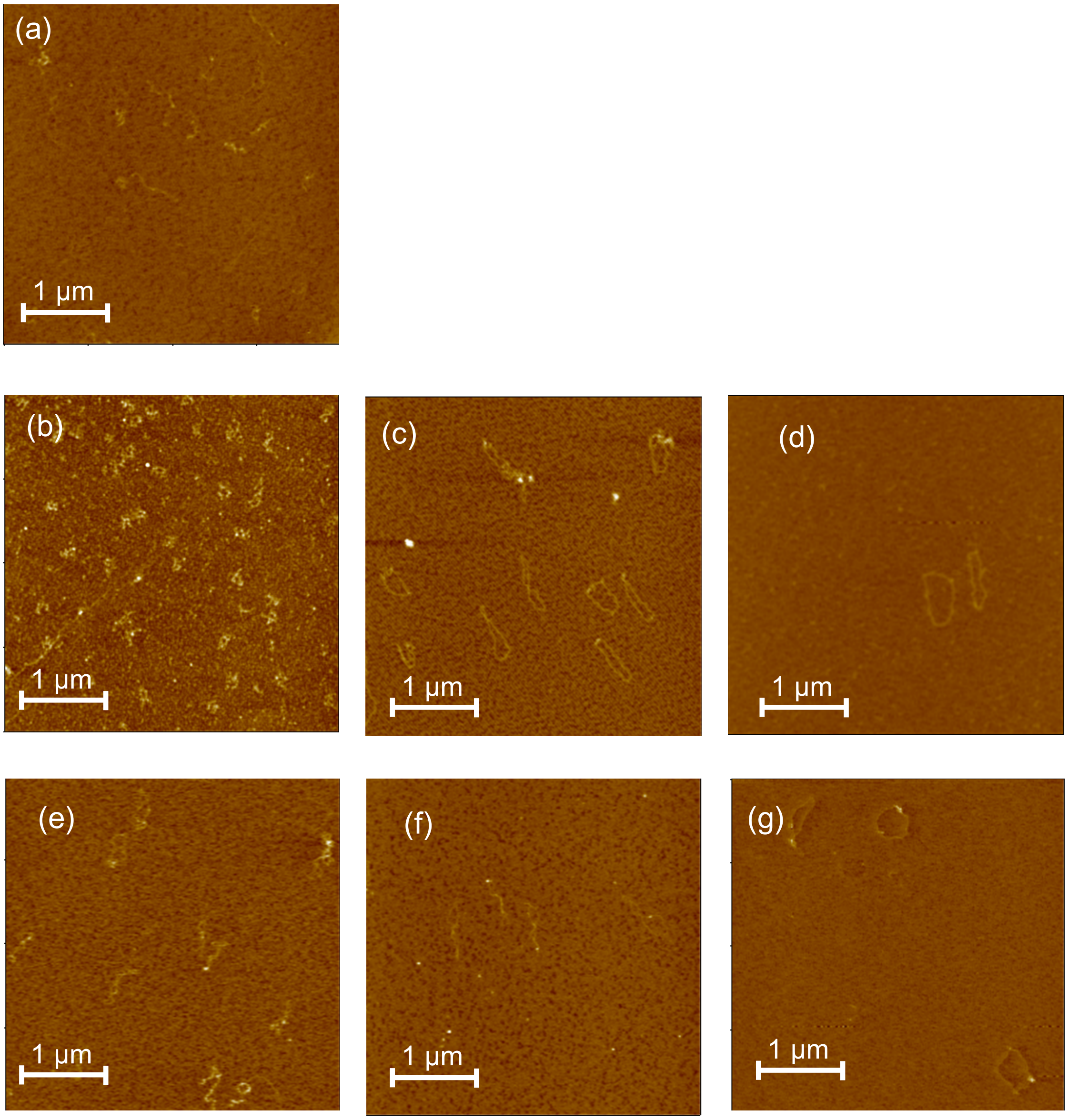


Figure S17 AFM images of (a) pDNA, pDNA mixed with BPy-Pt at BPy-Pt/pDNA base molar ratio of (b) 0.07, (c) 0.17 and (d) 0.23, and pDNA mixed with p(PEGMA-*co*-BPyMA-Pt) at Pt/P of (e) 0.07, (f) 0.17 and (g) 0.23.
